# XNAzymes targeting the SARS-CoV-2 genome inhibit viral infection

**DOI:** 10.1038/s41467-022-34339-w

**Published:** 2022-11-16

**Authors:** Pehuén Pereyra Gerber, Maria J. Donde, Nicholas J. Matheson, Alexander I. Taylor

**Affiliations:** 1 Cambridge Institute of Therapeutic Immunology & Infectious Disease (CITIID), Jeffrey Cheah Biomedical Centre, University of Cambridge, Cambridge, UK; 2 Department of Medicine, University of Cambridge, Cambridge, UK; 3 NHS Blood and Transplant, Cambridge, UK

**Keywords:** Catalytic DNA, Viral infection, Synthetic biology, Antisense oligonucleotide therapy, Nucleic acids

## Abstract

The unprecedented emergence and spread of SARS-CoV-2, the coronavirus responsible for the COVID-19 pandemic, underscores the need for diagnostic and therapeutic technologies that can be rapidly tailored to novel threats. Here, we show that site-specific RNA endonuclease XNAzymes – artificial catalysts composed of single-stranded synthetic xeno-nucleic acid oligonucleotides (in this case 2’-deoxy-2’-fluoro-β-D-arabino nucleic acid) – may be designed, synthesised and screened within days, enabling the discovery of a range of enzymes targeting SARS-CoV-2 ORF1ab, ORF7b, spike- and nucleocapsid-encoding RNA. Three of these are further engineered to self-assemble into a catalytic nanostructure with enhanced biostability. This XNA nanostructure is capable of cleaving genomic SARS-CoV-2 RNA under physiological conditions, and when transfected into cells inhibits infection with authentic SARS-CoV-2 virus by RNA knockdown. These results demonstrate the potential of XNAzymes to provide a platform for the rapid generation of antiviral reagents.

## Abstract (web summary)

RNA viruses have been responsible for large-scale epidemics and pandemics throughout the last few centuries. Here, the authors show the design, synthesis and screening of artificial RNA endonuclease XNAzymes capable of cleaving genomic SARS-CoV-2 RNA and self-assembling into enzymatic nanostructures inhibiting cellular viral replication.

## Introduction

Nucleic acid therapeutic technologies are proving to be crucial platforms for rapid response to emerging viral threats^1^. mRNAs encoding viral antigens for vaccination and monoclonal antibody therapies have joined a growing body of antiviral RNAi and antisense oligonucleotide (ASO) approaches^2–11^, as well as strategies involving CRISPR/Cas^12,13^, which may be rapidly engineered to target viral genomes and/or transcripts once their sequences are known. However, various challenges remain for these technologies, including issues of specificity, biostability, immunogenicity, target site accessibility, delivery, and the potential for viral mutational escape.

Modular nucleic acid catalysts capable of sequence-specific RNA transesterification – ribozymes and DNAzymes – offer an alternative platform for the generation of an almost unlimited variety of precision antivirals. However, despite the development of a range of variants of the classic ’10–23’ DNAzyme^14^ retargeted to viral RNAs^15–17^, including catalysts targeting the RNA genome of SARS-CoV coronavirus^18^, no antiviral DNAzyme has achieved clinical approval. A key concern is their dependence on unphysiological concentrations of divalent metal ions: the 10-23 catalytic core has been found to require high concentrations of magnesium (>10 mM) in order to fully fold into its active state^19^ whereas in intracellular free Mg^2+^ concentrations (0.5–1 mM) DNAzyme activity is severely reduced^20^. Although RNA knockdown effects can be measured in vivo when DNAzymes are exogenously delivered into cells, similar effects are observed in the absence of an active 10–23 catalytic core^21,22^, suggesting this is likely due to induction of RNase H (i.e. antisense mechanisms) and/or cytotoxic effects^21,23^.

Synthetic alternative bases, sugars and backbones (also known as xeno-nucleic acids, XNAs^24,25^) expand the capabilities of nucleic acid catalysts, either through modification of existing DNAzymes^26^ (e.g. ’X10-23’^27^, which has also been used to target SARS-CoV-2 RNA as the basis of a viral biosensor^28^) or the de novo evolution of fully-modified XNAzymes^29,30^. Recently, we described a modular XNAzyme, ’FR6_1’, composed of 2′-deoxy-2′-fluoro-β-d-arabino nucleic acid (FANA), originally selected to cleave a sequence in the RNA genome of the Zaire Ebolavirus, which has the ability to cleave long (>5 kb), structured RNA under physiological conditions in vitro and in vivo^31^.

RNA viruses have been responsible for numerous large-scale epidemics and pandemics throughout the 20th and 21st centuries, including catastrophic outbreaks of influenza, human immunodeficiency virus (HIV), coronavirus (SARS-CoV and MERS-CoV), flavivirus (Zika) and filovirus (Ebola) infection. Most recently, severe acute respiratory syndrome coronavirus 2 (SARS-CoV-2)^32^ has caused more than 600 million cases of coronavirus disease 2019 (COVID-19), over 6 million deaths worldwide^33^ and an estimated trillion-dollar reduction in productivity and economic growth. Despite the development of effective vaccines, waning immunity and the evolution of viral variants could mean SARS-CoV-2 will continue to pose a significant threat to human health for the foreseeable future^34,35^. Following the emergence of the first human cases in late 2019, there was a significant lag before the development of small molecule therapeutics such as the RNA-dependent RNA polymerase inhibitors remdesivir (Gilead) and molnupiravir (Merck), and variable efficacy in clinical trials^36,37^ suggest that continued discovery of novel antivirals and complementary modalities for tackling SARS-CoV-2 is necessary^38^. More broadly, the COVID-19 pandemic has underscored the need for expedited and streamlined drug discovery and development pipelines to prepare for future outbreaks^39^.

In cells infected with SARS-CoV-2, the 29,903 nt genomic RNA is used to produce at least ten canonical subgenomic RNAs, which together serve as transcripts encoding ~12 open reading frames (ORFs) and ~26 viral proteins, 16 of which are derived from two polyproteins produced by ribosomal slippage during translation of ORF1a and ORF1ab in the genomic RNA^40,41^. Thus far, therapeutic strategies have focused on the spike protein (S), which enables entry of the virus into the cell, the RNA-dependent RNA polymerase (RdRp) protein (nsp12), which copies the viral genome, the virally encoded proteases PL-PRO and 3CL-PRO (nsp3 and nsp5), the viral envelope protein E, the membrane protein M and the nucleocapsid protein N, which organises the genome in viral particles^42,43^. Direct targeting of the viral genome is an underexplored modality for the development of SARS-CoV-2 antivirals.

Here, we describe proof-of-concept design, testing and assembly of variants of the RNA endonuclease XNAzyme FR6_1, enabling the rapid development of a series of artificial enzymes and a biostable catalytic nanostructure targeting RNA sequences spanning the genome of SARS-CoV-2, and capable of inhibiting authentic viral infection in cells.

## Results

### Engineering XNAzymes to target SARS-CoV-2 RNA

The modular RNA endonuclease XNAzyme FR6_1 was initially selected to cleave an RNA sequence in the genome of Zaire Ebolavirus (’Sub_Ebo’) and is predicted to have an architecture comprised of a catalytic core flanked by two guide strands or substrate-binding ‘arms’^31^. Substrate specificity arises from a combination of the complementarity of the binding arms for target RNA, as well as putatively unpaired substrate residues positioned in a three-residue bulge opposite the catalytic core—although FR6_1 tolerates almost any dinucleotide pair (except GG) at the cleavage site in this bulge, there is an absolute requirement for an A one nucleotide downstream^31^. Initially, we searched the RNA genome of SARS-CoV-2 (NCBI NC_045512.2) for sequences with similarity to the original target sequence of FR6_1, but with no similar (i.e. off-target) sequences in the human transcriptome. Two sequences in SARS-CoV-2 open reading frame (ORF) 1b were identified, residues 14804–14822 (’Sub_ORF1b’) (61% homology with Sub_Ebo) and 13684–13718 (’Sub_ORF1b2’) (56% homology with Sub_Ebo). Although initial variants of FR6_1 targeted to these sequences (and prepared using FANA nucleotides and polymerase D4YK^44^) had little or no activity under quasi-physiological conditions (37 °C, 1 mM Mg^2+^, 150 mM KCl, pH 7.4) (Supplementary Fig. 1a), a screen of mutations in the XNAzyme core (Supplementary Fig. 1b, c) previously found to be beneficial for re-targeting FR6_1 to *KRAS* mRNA^31^, enabled the discovery of an improved ORF1b-targeting XNAzyme, ’Fz_CoV2_1b [A28G]’ (Supplementary Fig. 1d).

**Fig. 1:**
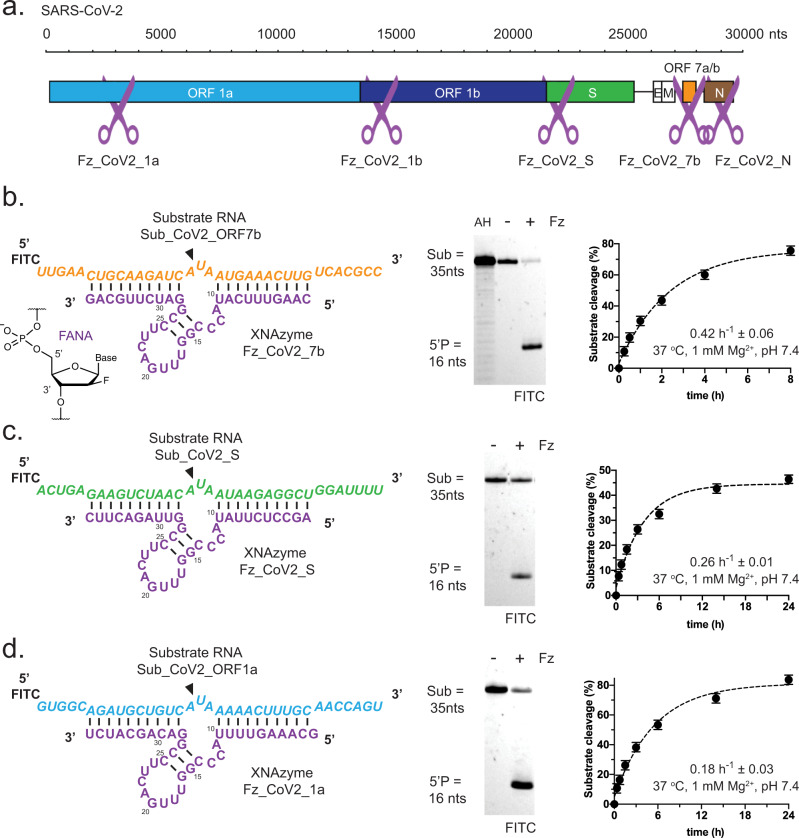
RNA endonuclease XNAzymes retargeted to the SARS-CoV-2 genome. **a** Schematic showing target locations in the SARS-CoV-2 RNA genome of all active XNAzymes described in this study (see also Supplementary Fig. 1). **b**–**d** Schematics showing sequences and putative secondary structures of XNAzymes targeting sequences in the **b** ORF7b, **c** Spike (S), or **d** ORF1a genes, bound to substrate RNAs containing their SARS-CoV-2 target sequences. Purple indicates FANA residues. Black arrows indicate the site of substrate cleavage. Urea-PAGE gels and graphs showing pseudo-first-order single-turnover reactions between (1 μM) RNA substrates and (5 μM) XNAzymes under quasi-physiological conditions (37 °C, 1 mM Mg^2+^, 150 mM KCl, pH 7.4) (gels show reactions after 15 h). AH indicates Sub_CoV2_ORF7b subjected to partial alkaline hydrolysis. Fz FANAzyme. Mean values ± SEM are shown for experiments performed in triplicate.

However, the catalytic rate of this XNAzyme on Sub_ORF1b was modest under quasi-physiological conditions (k_obs_ = 0.03 h^−1^ ± 0.01) (Supplementary Fig. 1e). We instead searched for potential RNA cleavage sites with the same five-nucleotide motif (5′ – CA^UAA – 3′, with ^ representing the site of cleavage) matching the bulge and flanking residues positioned for cleavage when the original FR6_1 XNAzyme is bound to its Ebola RNA substrate. This motif occurs 27 times in the SARS-CoV-2 genome; we synthesised RNA substrates analogous to four sites predicted by RNAfold^45^ to lie within regions of weak secondary structure in different ORFs, and screened corresponding retargeted FR6_1 XNAzymes under quasi-physiological conditions (Supplementary Fig. 1f), all of which showed measurable activity. We thus identified a total of five active XNAzymes with RNA target sites spanning the SARS-CoV-2 genome (Fig. 1a) but with no significant similarity to human RNAs. The three most active catalysts (Fig. 1b–d), targeting the regions encoding ORF7b (residues 27855–27877) (’Fz_CoV2_7b’) (Fig. 1b), the spike protein (residues 21850–21872) (’Fz_CoV2_S’) (Fig. 1c), and ORF1a (residues 2893–2915) (’Fz_CoV2_1a’) (Fig. 1d), were chosen for further investigation.

The XNAzymes were found to be highly specific endonucleases, able to cleave target SARS-CoV-2 RNA sequences in quasi-physiological conditions with single-turnover kinetics comparable to previous retargeted XNAzymes (*k*_obs_ = 0.18–0.42 h^−1^) (Fig. 1) and showed no activity on non-target SARS-CoV-2 RNA sequences in 48 h (Supplementary Fig. 2a). The XNAzymes retained their capacity for multiple-turnover catalysis, albeit with slow rates, performing 2–8 turnovers in 48 h (Supplementary Fig. 2b).

In order to benchmark the SARS-CoV2-targeting XNAzymes against other oligonucleotide catalysts, we also designed and synthesised a series of five analogous DNAzymes based on the 10–23 or 8−17 catalysts^14,46^, targeted to the same RNA substrate sequences, as allowed by the DNAzymes’ cleavage site preferences (Supplementary Fig. 3). Although these DNAzymes were found to efficiently cleave the 35 nt SARS-CoV2 RNAs in a high magnesium buffer (10 mM Mg^2+^) as expected, catalytic activities of all five DNAzymes were significantly reduced or abolished at quasi-physiological magnesium concentrations (1 mM Mg^2+^) and were clearly out-performed by the analogous XNAzymes (Supplementary Fig. 3).

Concerned that the SARS-CoV-2 nucleocapsid (N) protein, an RNA-binding protein responsible for genome packaging, could inhibit oligonucleotide catalysts, we next examined XNAzyme-mediated RNA cleavage under quasi-physiological conditions in the presence of N protein (up to 1.4 mM) (Supplementary Fig. 4). Activity of XNAzymes Fz_CoV2_7b, Fz_CoV2_S and Fz_CoV2_1a appeared to be unaffected, at least on short (35 nt) RNA substrates, even at concentrations reported to form condensates with nucleic acids (>10 μM)^47^ (Supplementary Fig. 4).

### Synthesis of a catalytic XNA nanostructure

**Fig. 2:**
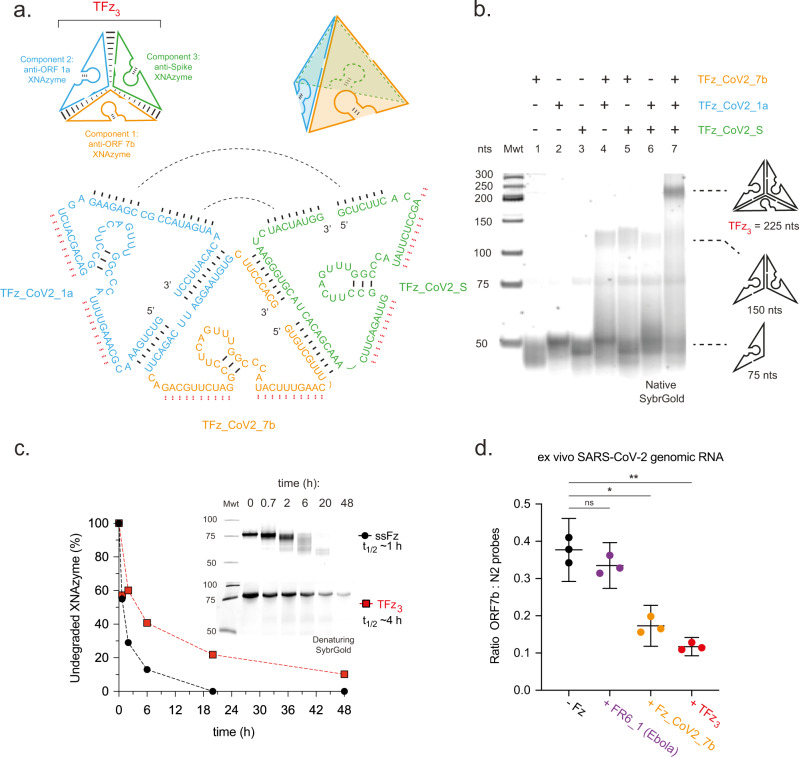
Assembling XNAzymes into a catalytic XNA nanostructure improves biostability whilst retaining the ability to cleave the genome of SARS-CoV-2. **a** Schematics showing the design of a three-component fully-FANA nanostructure (’TFz_3_’) presenting three different SARS-CoV-2 XNAzymes (Fz_CoV2_7b, Fz_CoV2_S and Fz_CoV2_1a) along single-stranded edges (strand hybridisation is designed to be mediated by base pairing indicated by black lines; red lines indicate residues mediating SARS-CoV-2 RNA substrate binding). **b** Native PAGE gel showing self-assembly of the complete TFz_3_ nanostructure is dependent on all three components. **c** Urea-PAGE gels and graph showing the stability of the purified, fully-assembled TFz_3_ (red squares) or a single-stranded component (’ssFz’: TFz_CoV2_7b)(black circles) in 50% human serum at 37 °C. **d** Quantification of cleavage of ex vivo SARS-CoV-2 genomic RNA determined by droplet digital RT-qPCR (ddPCR), measuring the target site of XNAzyme Fz_CoV2_7b in ORF7b relative to a non-target site (CDC N2 site in the Nucleocapsid (N) gene)(see Supplementary Fig. 6), following incubation (5 h) with nanostructure TFz_3_ (red circles), single XNAzyme Fz_CoV2_7b (orange circles), control XNAzyme FR6_1 (targeting Ebola RNA) (purple circles), or buffer alone (black circles), under quasi-physiological conditions (37 °C, 1 mM Mg^2+^, 150 mM KCl, pH 7.4). Horizontal bars and error bars represent mean values ± SEM for an experiment performed in triplicate and analysed using a two-tailed paired *t*-test (^ns^not significant *P* > 0.05, *P ≦ 0.05, **P ≦ 0.01). *P* values: −Fz vs FR6_1 = 0.3, −Fz vs Fz_CoV2_7b = 0.01, −Fz vs TFz_3_ = 0.005). The gels shown are representative of two independent experiments.

Next, we sought to combine SARS-CoV-2 XNAzymes in order to target the genome synergistically and to stabilise catalysts prior to examining activity in vivo. Previously, we found that a cocktail of three FR6_1-derived XNAzymes was able to cleave a long, structured RNA target at expected sites without mutual interference^31^. However, as FANA displays only modest resistance to serum exonucleases, further modifications (FANA phosphorothioate linkages) had to be introduced to improve biostability. An alternative approach is to sterically hinder the accessibility of 5′ and 3′ termini to exonucleases by incorporation into a nucleic acid nanostructure. We have previously shown that FANA is broadly compatible with DNA nanostructure designs^48^, so reasoned that an architecture used to improve DNAzyme stability by embedding catalysts into a simple self-assembling DNA polyhedron^49^ may be adapted to synthesise an analogous catalytic XNA nanostructure. We therefore appended additional FANA sequences on to Fz_CoV2_7b, Fz_CoV2_S and Fz_CoV2_1a, with regions of complementarity that enable assembly into a three-component 74.3 kDa nanostructure, ’TFz_3_’, in which each XNAzyme is presented on one of three single-stranded edges (Fig. 2a). As expected, all three components were necessary for assembly of the complete nanostructure, assayed by native PAGE (Fig. 2b), and activity of all three XNAzymes were retained (Supplementary Fig. 5a) with catalytic rates identical to those of the individual enzymes (Fig. 1 and Supplementary Fig. 5b). As unassembled components could be observed in TFz_3_ preparations (Fig. 2b), we further purified the 225 nts (74.3 kDa) TFz_3_ by depleting lower molecular weight species corresponding to monomeric and dimeric components (Supplementary Fig. 5c) and confirmed that the assembled nanostructure retained full activity (Supplementary Fig. 5d). As expected, the assembled TFz_3_ had an improved resistance to degradation in human serum (fourfold increase in half-life) compared with a single-stranded FANA component strand (Fig. 2c).

In the case of XNAzyme Fz_CoV2_S, the single-turnover reaction between the individual enzyme and the Sub_CoV2_S RNA plateaus at a lower level of cleavage (~45%) (Fig. 1c) than the reactions of other catalysts and their respective substrates (~80%)(Fig. 1b, d), presumably reflecting a greater tendency of this variant to fold into catalytically inactive conformations. However, the equivalent reaction between Sub_CoV2_S and the assembled TFz_3_ plateaus at ~60% (Supplementary Fig. 5b), is consistent with partial mitigation of misfolding by presentation in the nanostructure.

### Cleavage of SARS-CoV-2 genomic RNA in vitro

To evaluate the potential of the XNAzymes and the catalytic nanostructure to invade and cleave full-length (30 kb) genomic and/or subgenomic RNA, we next incubated the individual Fz_CoV2_7b XNAzyme, the assembled TFz_3_ nanostructure, or an irrelevant XNAzyme (the Ebola-targeting FR6_1 ‘parent’ FANAzyme) with ex vivo SARS-CoV-2 genomic RNA under single-turnover and quasi-physiological conditions (and the presence of ex vivo total cell RNA) (Fig. 2d). Cleavage was measured using a droplet digital RT-qPCR (ddPCR) assay specific for the target site (in ORF7b) with a non-target site (‘N2’) as reference (Supplementary Fig. 6a, b). After 5 h, relative depletion of amplicons corresponding to the cleavage site was clearly detected using both the single Fz_CoV2_7b enzyme (~60%) and the TFz_3_ nanostructure (~70%), but not control XNAzyme (FR6_1) (Supplementary Fig. 6c–e), comparable with the results obtained with short (35 nt) RNA substrates (Fig. 1b and Supplementary Fig. 5b). We have previously shown that it is critical to remove XNAzymes from RNA preparations when assessing RNA cleavage by RT-PCR as catalytic activity can occur during typical workups, as well as inhibition of target site RNA reverse transcription, yielding two sources of ‘false positive’ knockdown when measuring in vivo activity^31^. We, therefore, verified that our protocol for removal of FANAzymes also removed the TFz_3_ nanostructure by performing control assays after the addition of TFz_3_ and indeed observed no knockdown when the initial incubation step was omitted (Supplementary Fig. 6f–h).

### Inhibition of authentic SARS-CoV-2 infection

**Fig. 3:**
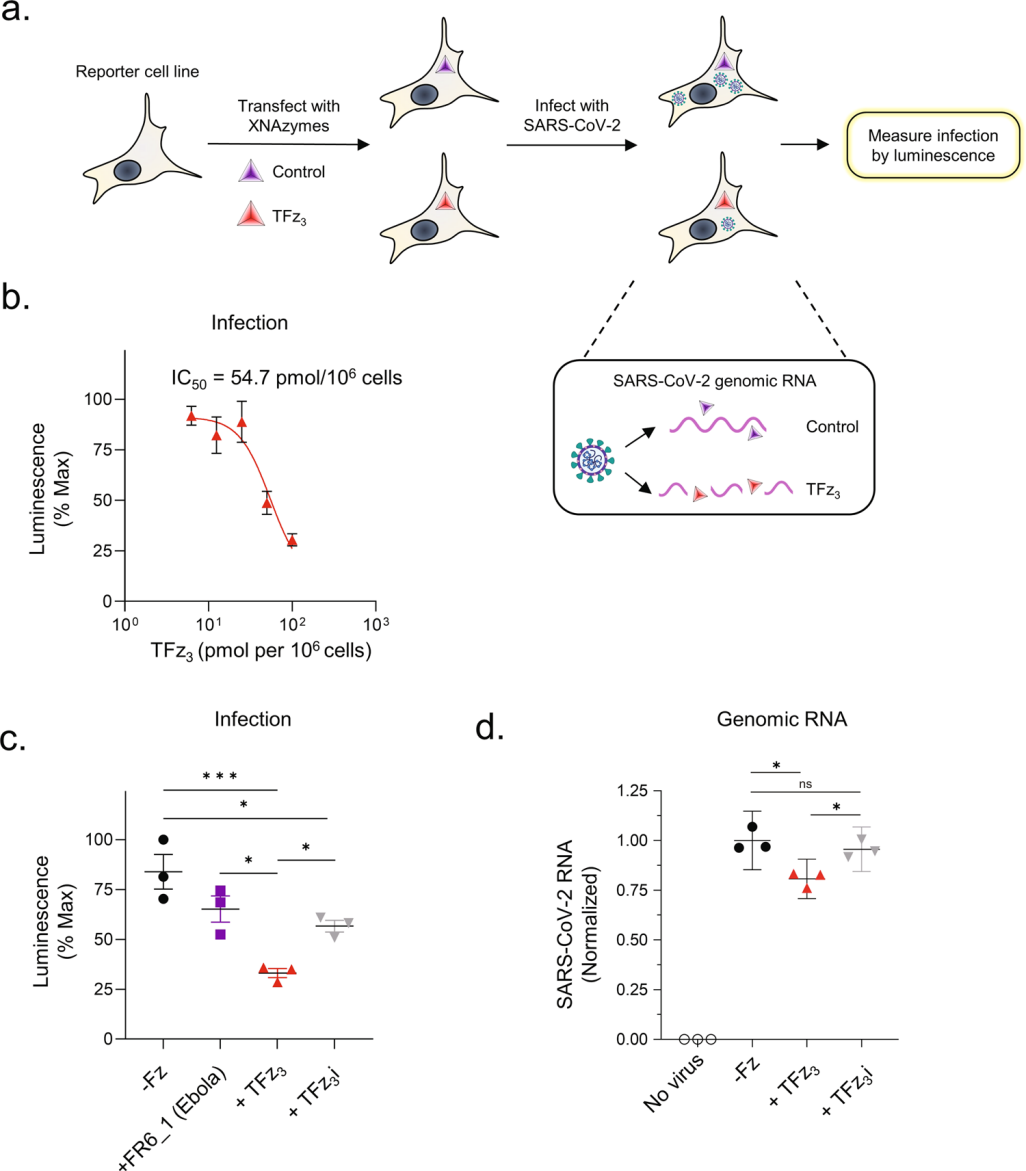
SARS-CoV-2 XNAzymes inhibit viral infection in cells. **a** Schematic showing the experimental design used to evaluate XNAzyme activity against SARS-CoV-2 infection. Luminescent HEK293T reporter cells^50^ express an inactive, firefly luciferase-based biosensor, activated by proteolytic cleavage during viral infection. **b** Inhibition of SARS-CoV-2 infection by the fully-FANA TFz_3_ catalytic nanostructure (’TFz_3_’). Reporter cells were transfected with the indicated doses of TFz_3_ (red triangles), then infected with SARS-CoV-2 at MOI = 0.01. Levels of infection were quantitated by luminometry after 16 h. Mean values ± SEM are shown for an experiment conducted in triplicate, representative of two independent experiments. **c** Specificity of inhibition by TFz_3_. Reporter cells were transfected with buffer alone (−Fz) (black circles) or (50 pmol per 10^6^ cells) control XNAzyme (’FR6_1’, targeting an RNA sequence from Ebola^31^) (purple squares), active TFz_3_ (red triangles), or a version of TFz_3_ containing catalytically inactive mutant XNAzymes (’TFz_3_i’) (grey triangles), then infected with SARS-CoV-2 at MOI = 0.01. Levels of infection were quantitated by luminometry after 16 h. Horizontal bars and error bars represent mean values ± SEM for an experiment conducted in triplicate, representative of two independent experiments. **d** Knockdown of SARS-CoV-2 RNA by TFz_3_. Reporter cells were transfected with buffer alone (−Fz) (black circles) or active TFz_3_(red triangles), or the catalytically inactive version (TFz_3_i) (grey triangles), then infected with SARS-CoV-2 at MOI = 0.01. Total RNA was extracted and purified 16 h post-infection, and genomic and/or subgenomic SARS-CoV-2 RNA (ORF7b cut site) was measured by droplet digital RT-qPCR (ddPCR), with normalisation to host cell *EIF2B2* mRNA. Horizontal bars and error bars represent mean values ± SEM for an experiment conducted in triplicate, representative of two independent experiments analysed using either **c** a one-way ANOVA and Dunnett’s test or **d** a two-tailed paired *t*-test (^ns^not significant *P* > 0.05, *P ≦ 0.05, **P ≦ 0.01, ***P ≦ 0.001), representative of two independent experiments. *P* values: **c** −Fz vs TFz_3_ = 0.0007, −Fz vs TFz_3_i = 0.03, FR6_1 vs TFz_3_ = 0.011, FR6_1 vs TFz_3_i = 0.6, TFz_3_ vs TFz_3_i = 0.049, **d** −Fz vs TFz_3_ = 0.02, −Fz vs TFz_3_i = 0.5, TFz_3_ vs TFz_3_i = 0.016.

Encouraged by in vitro activity of the SARS-CoV-2 XNAzymes under quasi-physiological conditions, we next asked whether the RNA genome could be targeted in vivo to inhibit infection with an authentic virus. As a proof-of-concept, we transfected the TFz_3_ nanostructure into reporter cells expressing a luminescent biosensor^50^, enabling quantitation of authentic SARS-CoV-2 infection (Fig. 3a). In these cells, cleavage of inactive, circularly permuted firefly luciferase by a viral protease (PL-Pro) during viral replication leads to an increase in luminescence, whilst co-expression of renilla luciferase allows normalisation for reporter levels and cell viability^50^. The luminescent signal reflects the frequency of infected cells, and, therefore, the rate of spreading infection over a 24 h time course. Transfecting with a series of concentrations of the TFz_3_ nanostructure, we observed a clear inhibition of SARS-CoV-2 replication in a dose-dependent manner (Fig. 3b) (luminescence reduced by up to 75% compared with cells transfected with buffer alone), with an indicative IC50 of 55 pmol/10^6^ cells. This was comparable to the IC50 observed when a version of the FR6_1 catalyst targeting *KRAS* G12D mRNA was transfected into RKO colon carcinoma cells (60 pmol/10^6^ cells)^31^, although in both cases, the final effective concentration of XNAzyme(s) inside cells was unknown. It is further unclear what proportion of the TFz_3_ nanostructures remain fully assembled inside cells, although the similar levels of XNAzyme activity between assembled nanostructures and unassembled components (Supplementary Fig. 5) suggest that any disassembly of TFz_3_ following transfection would affect XNAzyme biostability rather than catalytic performance directly. Although we did not explore other modifications of the FANA polymers in this study, we have previously shown that FANA XNAzymes can be further protected from exonucleases by phosphorothioate modifications, extending their half-life in serum by up to 16 h^31^.

To confirm the specificity of the inhibitory effect, we first transfected cells with an irrelevant XNAzyme (FR6_1, targeting Ebola RNA). As expected, this abrogated the inhibition of SARS-CoV-2 infection seen with the TFz_3_ nanostructure (Fig. 3c). Since differences in biostability between individual FR6_1 and nanostructure-embedded TFz_3_ XNAzymes (Fig. 3c) could influence these observations, we next sought to develop a version of the TFz_3_ nanostructure in which all three XNAzymes are catalytically inactive (’TFz_3_i’) as an additional control. We had previously found that a single point mutation in the FR6_1 catalytic core, fG27fA (using numbering derived from the parental XNAzyme), was sufficient to render the XNAzyme catalytically inactive without significantly altering substrate binding^31^. We, therefore, synthesised equivalent [fG27fA] mutant versions of all three components of the nanostructure (Supplementary Fig. 7a), used these to assemble TFz_3_i (Supplementary Fig. 7b), and verified that this version was indeed catalytically inactive (Supplementary Fig. 7c). Although a small inhibition of SARS-CoV-2 infection was observed when cells were transfected with the inactive TFz_3_i nanostructure (Fig. 3c)(20–30% compared with cells transfected with buffer alone), active XNAzyme catalytic cores were confirmed to be necessary for the full inhibitory effect.

### Mechanism of SARS-CoV-2 inhibition

To better understand the mechanism of XNAzyme inhibition of SARS-CoV-2 infection, we examined whether the effect was accompanied by specific knockdown of SARS-CoV-2 genomic and/or subgenomic RNA. Total RNA was extracted from cells infected with authentic SARS-CoV-2 following transfection with the TFz_3_ nanostructure, inactive TFz_3_i, or buffer alone. Viral RNA was measured by ddPCR (using hydrolysis probes binding to the ORF7b target site of one of the XNAzymes in the TFz_3_ nanostructure, TFz_CoV2_7b) with normalisation to host cell *EIF2B2* mRNA (Fig. 3d). Consistent with the inhibition of SARS-CoV-2 infection observed using the luminescent biosensor, SARS-CoV-2 RNA knockdown (~25% cleavage of the ORF7b site) was apparent in cells transfected with active TFz_3_ (which cleaves sites in ORFs S and 1a in addition to 7b)_,_ but not inactive TFz_3_i.

**Fig. 4:**
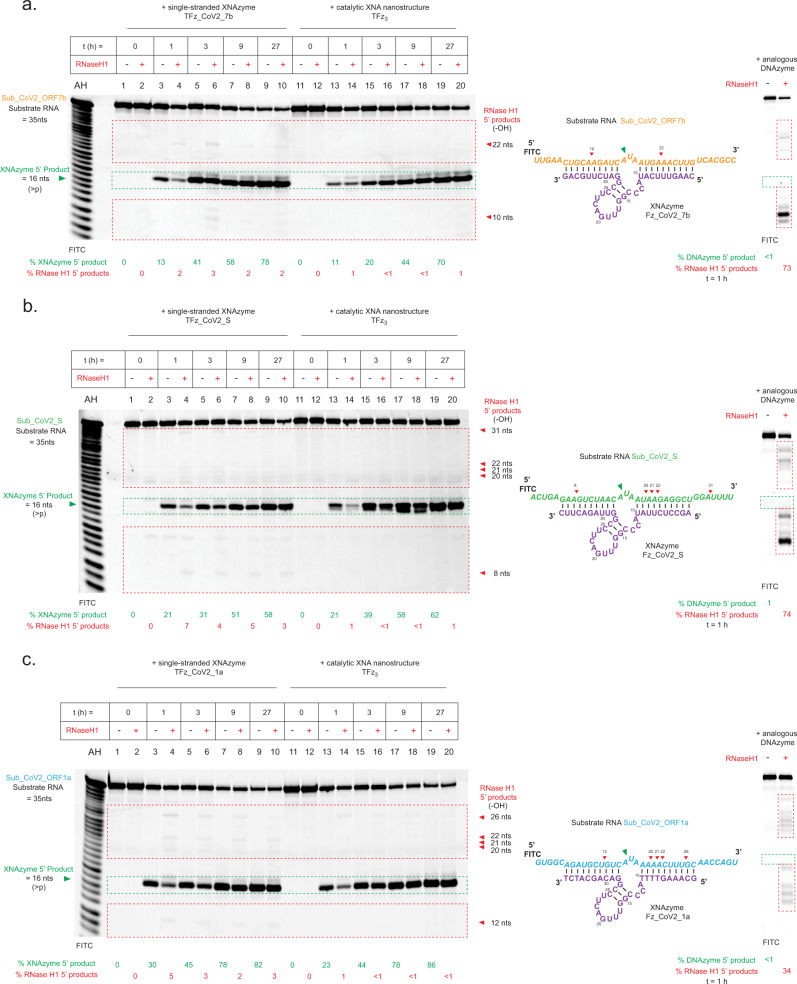
Assembling XNAzymes into a catalytic XNA nanostructure limits RNase H1-mediated RNA cleavage. **a**–**c** Urea-PAGE gels showing human RNase H1 assays and schematics showing deduced RNA products and cleavage sites mediated by RNase H1 (indicated by red dotted line boxes and red arrows) or mediated by XNAzyme catalysis (indicated by green dotted line boxes and green arrows). Human RNase H1 was incubated with (0.25 μM) RNA substrates (**a**) Sub_CoV2_ORF7b, (**b**) Sub_CoV2_S or **c** Sub_CoV2_ORF1a, and either (0.25 μM) single-stranded component XNAzymes, purified fully-assembled nanostructure (TFz_3_) or an analogous DNAzyme targeting the same substrates: **a** Dz817_CoV2_7b, **b** Dz817_CoV2_S or **c** Dz1023_CoV2_1a (See also Supplementary Figs. 3, 8) (37 °C, 1 mM Mg^2+^, 20 mM KCl, pH 7.5). Note that partial alkaline hydrolysis of RNA substrates (AH), as well as cleavage mediated by the FR6_1 XNAzyme catalytic core, produce 5′ RNA products that terminate in 3′ cyclic phosphate (>p)^31^, whereas RNase H1-mediated cleavage produces 3′ OH termini, resulting in a difference in PAGE mobility equivalent to one nucleotide. The gels shown are representative of two independent experiments.

Interestingly, we had expected the catalytically inactive nanostructure TFz_3_i to produce a measurable knockdown effect despite the fG27fA mutation(s), due to the potential for FANA-containing oligonucleotides to trigger RNase H-dependent cleavage of viral RNA^51^ (i.e. ‘antisense’-mediated knockdown), which can contribute to knockdown induced by FANAzymes, but is generally less specific^31^, and would be consistent with the small inhibitory effect of TFz_3_i observed in the infection assay (Fig. 3c). To explore this further, we mapped potential RNase H1 cleavage sites induced by the individual XNAzymes and the active and inactive nanostructures in vitro and compared these with RNase H1 activation by analogous DNAzymes (Supplementary Fig. 8). Although expected RNase H1-mediated cleavage induced by the substrate-binding arms could be observed with all three individual XNAzymes and their inactive variants, RNase H1 recruitment was 7–10-fold lower than by DNAzymes and appeared to be further reduced by assembly into the nanostructure. To confirm this, we directly compared timecourses of RNA cleavage dependent or independent of RNase H1 with either single-stranded component XNAzymes or the purified fully-assembled TFz_3_ catalytic nanostructure (Fig. 4). Assembly into the nanostructure indeed reduced the capacity of component XNAzymes to trigger RNase H1 (at least in vitro), resulting in bona fide XNAzyme catalytic turnover yielding the majority of RNA cleavage (≤1% RNase H1-mediated cleavage vs 60–90% XNAzyme-mediated cleavage after 27 h) (Fig. 4). Presumably, the architecture of the nanostructure restricts RNase H1 access to the RNA x FANA heteroduplex formed by substrate binding along the edges of the nanostructure—consistent with the observation that the catalytically-inactive TFz_3_i, despite containing complete substrate-binding arms of all three XNAzymes (Supplementary Fig. 7) showed significantly less inhibition of the virus than the active TFz_3_ (Fig. 3c) and induced little to no genomic RNA knockdown (Fig. 3d)—the observed SARS-CoV-2 inhibition by the active TFz_3_ nanostructure therefore appears to be dependent on direct cleavage of the genomic RNA via catalytic turnover of the embedded XNAzymes, independent from antisense-type effects, in order to achieve the full effect.

The discrepancy between the small inhibitory effect observed with the inactive TFz_3_i nanostructure in the infection reporter assay (Fig. 3c) and the apparent absence of viral RNA cleavage (Fig. 3d) suggests some component of inhibition independent of catalytic activity. One possibility is FANA interaction with viral or host cell proteins, reducing or inhibiting the viral protease without reduction of viral RNA. Alternatively, the level of RNase H1-mediated cleavage induced by TFz_3_i at the single site in ORF7b (as the RT-qPCR assay measures) may simply be too small (<10%) to measure accurately—whereas, in the reporter assay, inhibition derived from this effect would be the result of simultaneous RNase H1-induction across all three SARS-CoV-2 RNA sites targeted by the three components of TFz_3_i.

## Discussion

Here we report the design, synthesis and screening of a series of RNA endonuclease XNAzymes specific for five sites across the genome of SARS-CoV-2 with a cumulative turnaround time for a single researcher of less than 1 week (although delays occurred in real time due to supply interruptions during the first wave of the pandemic). Although the retargeted enzymes exhibited variable catalytic rates which were lower than that of the parental FR6_1 XNAzyme (and in one case, ’Fz_CoV2_1b2’, no activity was detectable (Supplementary Fig. 1a)), three XNAzymes were sufficiently active in physiological conditions to demonstrate proof-of-concept knockdown of SARS-CoV-2 genome in vitro and in vivo, reducing viral infection by about 75% at the highest concentration transfected (100 pmol per 10^6^ cells), with 60–70% of this effect attributable to specific XNAzyme activity (Fig. 3c). In the context of other nucleic acid technologies targeting SARS-CoV-2, the XNAzymes thus compare favourably (with the caveat that inhibition is reported using disparate assays): an antiviral microRNA exhibited 28% inhibition^52^ and initial siRNAs targeted to the spike, nucleocapsid or membrane genes generally yielded 40–60% reduction in viral levels^8^. Although improved siRNAs have been discovered by extensive target selection and screening, similar levels of inhibition were seen with most of the top candidates (8 of 11 siRNAs reported by ref. 11 showed 25–80% inhibition, 12 of 18 reported by ref. 5 showed ≤50% inhibition, and 5 of 8 reported by ref. 10 showed <65% inhibition). Likewise, in a CRISPR-Cas13-based strategy, the majority of crRNA guide strands screened (32 of 40), yielded ≤50% inhibition^13^. The hit rates in these studies suggest that a broader screen of XNAzyme target sites, and optimisation of re-programmed catalysts, could yield XNAzymes with improved inhibition.

We have previously noted that FR6_1 exhibits sequence preferences at the site of substrate cleavage^31^, and indeed the three most active retargeted catalysts, Fz_CoV2_7b, Fz_CoV2_S and Fz_CoV2_1a, were obtained when preserving the original 5′ – CA^UAA – 3′ motif^31^ (derived from the target site in the Zaire Ebolavirus genome) opposite the catalytic core. However, the variability in rates and cleavage plateaus between these XNAzymes suggests activity is also impacted by other sequences or secondary structures in their respective substrates and/or substrate-binding arms. One explanation could be differences in the stability of alternative RNA x FANA binding modes between XNAzyme variants - i.e. interactions between the substrate and core residues that could enable the core to adopt misfolded conformations, such that each XNAzyme has a distinct set of conformers in equilibrium, with different proportions of catalytically active and inactive folds. Consistent with this, embedding XNAzymes into the nanostructure design improved the proportion of maximal substrate cleaved (in the case of Fz_CoV2_S), presumably by constraining substrate binding arms into extended conformations, favouring the catalytically active binding mode. Alternatively, differences in target accessibility due to substrate secondary structure may also impact rates. The comparatively slow rates of multiple turnover observed with previous variants of the FR6_1 XNAzyme^31^ and here (Supplementary Fig. 2) (in part as we made no attempt to optimise binding arm length in this study) suggest that the trade-off between strand invasion of structured substrates and subsequent product inhibition continues to be a limiting factor for XNAzymes and oligonucleotide catalysts in general. Nonetheless, in applications where target RNAs are present in comparatively low copy number and/or are long-lived, knockdown may nonetheless be achievable at reasonable doses even when catalysts perform few turnovers, as here. In the first few hours post-infection, SARS-CoV-2 genomic RNA is typically present at 10–100 copies per cell and has a half-life of 6–8 h^53^, suggesting synthesis could, in principle, be outpaced by antiviral XNAzymes delivered at picomolar concentrations with k_obs_ as slow as 0.1 h^−1^. Although potentially time-consuming, some optimisation of retargeted XNAzymes in order to achieve further rate gains is nonetheless possible by directed evolution^29,31^ and may be necessary for applications where superior inhibitors are required.

Future efforts could be directed toward; (1) improving methods for selecting optimal cleavage sites within long target RNAs - whilst we did not exhaustively screen sites within the SARS-CoV-2 genome in this study, in vitro selection for optimal sites is feasible^54,55^, and could be informed by knowledge of siRNA efficacy^9,56^ and genomic architecture^57^—it may be of interest to explore whether XNAzymes can target negative sense genomic RNA, which appears to be inaccessible by RNAi^9^; (2) understanding the structure of the XNAzyme core, and XNA nanostructure assembly, in greater detail in order to optimise the folding landscape of retargeted enzymes; (3) improving turnover, perhaps through systematic evaluation of modifications to substrate-binding arms or other strategies^58^ and (4) extension of XNAzyme selection to alternative chemistries with beneficial physicochemical properties, such as ‘locked nucleic acids’ (LNAs), a chemistry used to develop ‘gapmer’ ASOs capable of 70–90% inhibition of SARS-CoV-2^6^. It may also be possible, for example, to engineer XNAzymes from nucleotide analogue building blocks that are themselves antiviral^2^ in order to derive therapeutic efficacy from both the activity of the functional oligonucleotide but also the products of its degradation in vivo^59^.

Optimisation of XNAzyme delivery is also a key outstanding problem. Here, we limited our study to the transfection of reporter cells in order to establish XNAzyme antiviral activity in vivo. It is reasonable to suggest that existing methods of delivery of functional oligonucleotides (e.g. lipid nanoparticles, polymer formulations, cell-penetrating peptides or aptamers^60,61^) will be adaptable to XNAzymes. Naked (gymnotic) delivery may also be possible, as shown for FANA antisense oligos^62^ and DNAzymes targeting RNA in the lung^63^. In this regard, nanostructures may offer enhanced uptake^64^. However, such approaches must contend with the need to deliver comparatively large doses when catalysts have limited capacity for multiple turnover. Furthermore, it is unclear whether viral genomic RNA, which is transcribed within double-membrane replication organelles^65^, would be accessible at all stages of viral infection and replication without, for example, conjugation to amphipathic compounds^60^.

Our results nonetheless demonstrate that the cleavage capabilities of XNAzymes can be extended to genomic RNA and establish knockdown without substantial involvement of host effectors (such as RNase H), which we had previously found contribute to in vivo activity^31^, but here were limited by a presentation of XNAzymes in a nanostructure, in principle boosting the specificity of this approach. Unlike other nucleic acid technologies such as RNAi, XNAzymes thus have the capacity to circumvent dependence on host factors for activity, and overcome the activity limitations of previous oligo catalysts, as we demonstrate for 10–23 and 8–17 DNAzymes. As well as the potential for reduced disruption of host regulatory networks^66^, the strategy we describe may also avoid viral mechanisms for blocking silencing machinery^67^ and, in principle, enable RNA knockdown to be extended to subcellular compartments or extracellular vesicles in which host effectors are scarce or unavailable, such as replication organelles or exosomes, of particular relevance due to their role in the cell-cell transfer of viral components^68^. Although a more thorough evaluation of their potential for off-target effects is necessary, the enhanced specificity of oligo catalysts also suggests that targeting multiple RNA sequences using a cocktail of XNAzymes or a multifunctional construct (as we demonstrate here) ought to be a feasible general strategy to avoid the emergence of escape variants observed when single sites in genomic RNA of rapidly mutating RNA viruses are targeted by siRNAs, for example^69^.

Our findings demonstrate the potential for modular RNA endonuclease XNAzymes to provide a platform technology for the rapid discovery of specific antiviral reagents. Although further development of catalyst designs and chemical composition are likely necessary for the clinical impact of this technology, as well as a more detailed understanding and optimisation of intracellular activity and pharmacokinetics, XNAzymes are well-placed to join the growing toolbox of nucleic acid therapeutics and technologies in preparation for rapidly emerging biological threats.

## Methods

DNA and RNA (and chimeric DNA/RNA) oligonucleotides were synthesised by Integrated DNA Technologies (Belgium) or Sigma-Aldrich/Merck (USA). Polymerase D4K^44^ was kindly provided by Philipp Holliger (MRC Laboratory of Molecular Biology, Cambridge). Recombinant SARS-CoV-2 nucleocapsid (N) protein (residues 48-365 with N-terminal His-tag, expressed in *E. coli*) was kindly provided by Jakub Luptak and Leo James (MRC Laboratory of Molecular Biology, Cambridge). Ex vivo genomic SARS-CoV-2 RNA (purified from infected Vero cells) was obtained from Launch Diagnostics, UK (Vircell Amplirun coronavirus SARS-CoV-2 RNA control MBC137-R). HEK293T cells (authenticated by STR profiling) were kindly provided by Paul Lehner (University of Cambridge) and modified to generate the SARS-CoV-2 reporter cell line as detailed in ref. 50. VeroE6 cells (authenticated by species-specific PCR (IDEXX BioAnalytics)) were kindly provided by Rupert Beale (Francis Crick Institute).

The SARS-CoV-2 virus used in this study was the lineage B viral isolate SARS-CoV-2/human/Liverpool/REMRQ0001/2020, a kind gift from Ian Goodfellow (University of Cambridge), isolated by Lance Turtle (University of Liverpool), David Matthews and Andrew Davidson (University of Bristol) from a patient from the Diamond Princess cruise ship^70–72^.

Detailed characterisation of the FR6_1 FANA XNAzyme catalyst is provided in ref. 31 and verification of sequence fidelity of FANA oligonucleotides prepared using polymerase D4K is provided in ref. 44.

### Preparation of XNAzymes and RNA substrates

FANA oligonucleotides were synthesised by polymerase extension reactions using triphosphates of FANA (faNTPs) (Metkinen Chemistry, Finland) and polymerase D4K^29,30^, templated by 3′ biotinylated DNA templates (e.g. ’Fz_CoV2_7b_temp’; Supplementary Table 1) and primed using the chimeric DNA/RNA primer ’drP2_Ebo’. Synthesised strands were isolated from templates by incubating reactions with streptavidin magnetic beads (Dynabeads MyOne C1, Thermo Fisher Scientific, USA) and eluting using 0.1 N sodium hydroxide. FANA oligos were treated with 0.7 N NaOH for 1 h at 65 °C to remove DNA/RNA primers by hydrolysis. All XNAzymes and RNA substrates (except genomic SARS-CoV-2 RNA) were purified by denaturing urea-PAGE and desalted by ethanol precipitation. Prior to initiation of RNA cleavage reactions, XNAzymes and RNA substrates were annealed in nuclease-free water (Qiagen, Germany) by incubation at 80 °C for 60 s, then cooled to room temperature for 5 min.

Partially hydrolysed RNA substrates (for use as electrophoresis size standards) were prepared by incubation at 65 °C in 20 mM sodium hydroxide (pH 12) for 4 min, then neutralised with 1 M Tris pH 7.

### Determination of XNAzyme and DNAzyme activity on short substrates

PAGE-purified XNAzymes (5 μM) or DNAzymes (5 μM), and short (35 nt) RNA substrates (1 μM) were annealed separately as described above and reacted at 37 °C in either quasi-physiological buffer (30 mM EPPS pH 7.4, 150 mM KCl, 1 mM MgCl_2_) or high magnesium buffer (30 mM EPPS pH 7.4, 150 mM KCl, 10 mM MgCl_2_). Reactions were stopped by the addition of excess PAGE gel loading buffer (95% formamide, 20 mM Tris pH 7.5, 10 mM EDTA, 0.05% bromophenol blue) and analysed by urea-PAGE using 20% acrylamide gels. Fluorophore-labelled RNA substrates were visualised using an FLA-5000 scanner (Fujifilm, Japan) and quantified using ImageQuant TL (GE Healthcare) or Fiji^73^. Pseudo-first-order reaction rates (*k*_obs_) under single-turnover pre-steady-state (*K*_m_/*k*_cat_) conditions were determined from timecourses; reactions were sampled and stopped at appropriate intervals by snap-freezing on dry ice in excess PAGE gel loading buffer and analysed as above. Quantification data from three independent replicates per time course were fit to a one-phase association model using Prism 9 (GraphPad)^30^.

### XNA nanostructure synthesis and assembly

The sequences for the FANA nanostructure were adapted from components of a DNA design described by ref. 49 (hybridising sequences were derived from components ’S3’, ’S4’ and ’S5’ in ref. 49). FANA component strands for the active nanostructure (’TFz_3_’), ’TFz_CoV2_7b’, ’TFz_CoV2_S’ and ’TFz_CoV2_1a’, or the inactive nanostructure (’TFz_3_i’), ’TFz_CoV2_7b_i’, ’TFz_CoV2_S_i’ and ’TFz_CoV2_1a_i’, were prepared using the relevant DNA templates (Supplementary Table 1) and drP2_Ebo primers, which were subsequently removed by hydrolysis as described above and PAGE-purified. Nanostructure component strands were mixed in an equimolar ratio in nuclease-free water (Qiagen, Germany) and annealed by incubation at 95 °C for 2 min, then cooled to 15 °C at 0.1 °C/sec. Nanostructures were incubated at 37 °C for 1 h in quasi-physiological buffer (30 mM EPPS pH 7.4, 150 mM KCl, 1 mM MgCl_2_), then purified using 50KDa ultra centrifugal filter concentrator (Amicon) as recommended by the manufacturer, with three 10 min wash steps with the same buffer.

### Biostability assay

TFz_3_ nanostructure or one of the single-stranded components (TFz_CoV2_7b) were annealed and purified as described above, then incubated (at 5 μM final) at 37 °C in 50% human serum (Sigma-Aldrich/Merck) for 48 h. Reactions were sampled and stopped at appropriate intervals by snap-freezing on dry ice in excess PAGE gel loading buffer. The proportion of undegraded full-length catalyst was determined by Urea-PAGE; gels were stained with SYBR Gold (Thermo Fisher Scientific, USA) and imaged as described above.

### Determination of XNAzyme activity on ex vivo SARS-CoV-2 genomic RNA

PAGE-purified XNAzymes (0.5 μM) or TFz_3_ nanostructure (0.5 μM each component, annealed without further purification) and incubated with ex vivo genomic SARS-CoV-2 RNA (~3000 copies/μl) in quasi-physiological buffer supplemented with 50 ng/μl total eukaryotic cell RNA (purified from RKO cells) and RNasein (1 U/μl)(Promega, USA) for 5 h at 37 °C. Reactions were purified using an RNeasy kit (Qiagen, Germany)) with additional RPE washes to remove XNAzymes (see ref. 31). Eluted RNA was reverse transcribed using Advanced iScript RT (Bio-Rad Laboratories, USA) with random primers, according to the manufacturer’s instructions. 3 ul of the RT reaction was used in a 20 μl emulsion PCR for cDNA quantification by Droplet Digital PCR (ddPCR), prepared using ddPCR Supermix, Droplet Generation Oil and a QX-200 system (Bio-Rad Laboratories, USA), according to the manufacturer’s instructions.

Specific cleavage at the target site for XNAzyme FzCoV2_7b in ORF7b (residues 27873–27897 in the SARS-CoV-2 reference sequence (NC_045512.2)) was quantitated by ddPCR, and parallel reactions performed for normalisation to a non-target site in the N-protein gene (the CDC ‘N2’ site (https://www.cdc.gov/coronavirus/2019-ncov/lab/rt-pcr-panel-primer-probes.html), residues 29188– 29210) using singleplex hydrolysis probe-based assays (performed in triplicate): 0.5 μM primers (’nCoV_ORF7b_Fw’ and ’nCoV_ORF7b_Rev’ for the ORF7b assay, or ’nCoV_N2_Fw’ and ’nCoV_N2_Rev’ for the N2 assay) and 0.125 μM probe ('nCoV_ ORF7b _Probe' for the ORF7b assay, or 'nCoV_N2_Probe' for the N2 assay) were used with the following cycling conditions: 95 °C 10 min, 40 x [94 °C for 30 s, 58 °C for 1 min (with 2 °C/s ramp rate)], 98 °C for 10 min. Data were analysed using Quantasoft Analysis Pro software (Bio-Rad Laboratories, USA) and Prism 9.2.0 (Graphpad Software, USA). Statistical significance was determined using a paired *t*-test.

### Cell culture and XNAzyme transfection

HEK293T SARS-CoV-2 reporter cells^50^ were cultured in DMEM supplemented with 10% foetal calf serum (FCS), 100 units/ml penicillin, and 0.1 mg/ml streptomycin at 37 °C in 5% CO_2_. Cells were regularly screened and confirmed to be mycoplasma negative (Lonza MycoAlert and IDEXX BioAnalytics). PAGE-purified XNAzymes, TFz_3_ nanostructures (annealed without further purification), DNAzymes, siRNA or buffer alone were transfected into cells growing at ~60% confluency (detached using 0.25% trypsin EDTA) using a Neon electroporation system (Thermo Fisher Scientific, USA). 800,000 cells were used per transfection with 10 ul Neon tips and 2 × 20 ms pulses of 1400 V.

### Production and titration of SARS-CoV-2 viral stocks

Viral stocks were prepared by passaging once in VeroE6 cells. In brief, VeroE6 cells were infected at a low multiplicity of infection (MOI) with the original viral stock and incubated for 72 h (by which time the cytopathic effect was evident). Virus-containing culture supernatants were then clarified by centrifugation at 600 × *g* for 5 min, and immediately frozen in aliquots at −80 °C. For the determination of MOIs, viral stocks were titrated in VeroE6 cells by 50% tissue culture infectious dose (TCID50) assays using standard methods.

### Quantitation of SARS-CoV-2 infection

Quantification of SARS-CoV-2 replication in HEK293T reporter cells is detailed in ref. 50. In brief, HEK293T reporter cells over-expressing ACE2, Renilla luciferase (Rluc) and SARS-CoV-2 Papain-like protease-activatable circularly permuted firefly luciferase (FFluc) were transfected with XNAzymes (as above), then seeded in flat-bottomed 96-well plates at 50,000 cells per well. Cells were allowed to attach for 4 h and then infected with SARS-CoV-2 at MOI = 0.01. After 16 h, cells were lysed in Dual-Glo Luciferase Buffer (Promega) diluted 1:1 with PBS and 1% NP-40. Lysates were then transferred to opaque 96-well plates, and viral replication was quantitated as the ratio of FFluc/Rluc activity measured using the Dual-Glo kit (Promega) according to the manufacturer’s instructions. FFluc/Rluc ratios were expressed as a percentage of the maximum. Where indicated, the IC50 was obtained using the Sigmoidal, 4PL, X is log(concentration) function in GraphPad Prism. Statistical significance was determined using a one-way ANOVA followed by Dunnett’s multiple comparison test.

### Quantification of in vivo SARS-CoV-2 RNA

Total RNA from HEK293T SARS-CoV-2 reporter cells treated as above was extracted following lysis in RNeasy RLT buffer (Qiagen) supplemented with 2-mercaptoethanol (10 μl/ml) and purified using RNeasy kits (Qiagen, Germany with on-column DNase digestion according to the manufacturer’s instructions, and additional RPE washes in order to fully remove FANA oligos (see ref. 31 for a discussion of the necessity of this precaution), and reverse transcribed to generate cDNA templates for ddPCR reactions as in 'Determination of XNAzyme activity on ex vivo SARS-CoV-2 genomic RNA'. Quantification of SARS-CoV-2 RNA (ORF7b and N2 sites) was performed in triplicate and normalised to a host reference gene, *eIF2B2*, using a singleplex assay: 0.9 μM primers ('eIF2B2_Fw' and 'eIF2B2_Rev') and 0.5 μM probe ('eIF2B2_Probe') were used with the following cycling conditions: 95 °C 10 min, 40 x [94 °C for 30 s, 58 °C for 1 min (with 2 °C/s ramp rate)], 98 °C for 10 min. Data were analysed using Quantasoft Analysis Pro software (Bio-Rad Laboratories, USA) and Prism 9.2.0 (Graphpad Software, USA). Statistical significance was determined using a paired *t*-test.

### Determination of XNAzyme-induced RNase H1 cleavage

Human RNase H1 assays were performed by incubating co-annealed RNA substrates (0.25 μM) and XNAzymes (0.25 μM) with 0.01 ug/ul recombinant human RNase H1 (RayBiotech, USA) in 20 mM Tris-HCl (pH 7.5), 20 mM KCl, 5 mM DTT, 1 mM MgCl_2,_ for 1 h at 37 °C. Reactions were stopped by the addition of an excess of formamide PAGE loading buffer and snap-frozen on dry ice. Reactions were analysed by urea-PAGE, using 20% acrylamide gels. Fluorophore-labelled RNA substrates were visualised using an FLA-5000 scanner (Fujifilm, Japan) and quantified using ImageQuant TL (GE Healthcare) or Fiji^73^.

### Reporting summary

Further information on research design is available in the Nature Research Reporting Summary linked to this article.

## Supplementary information

Supplementary Information

Reporting Summary

## Data Availability

All the data generated in this study are available within the main text and the Supplementary Information file. Data are also available from the corresponding author upon request. NCBI SARS-CoV-2 genome reference sequence: NC_045512.2. Source data are provided with this paper.

## Source data

Source Data
