# Supplementary material for: XNAzymes targeting the SARS-CoV-2 genome inhibit viral infection
Source: Nat Commun. 2022 Nov 16;13:6716. doi: 10.1038/s41467-022-34339-w (PMC9668987; doi:10.1038/s41467-022-34339-w)
Supplement: Supplementary file 1 — Supplementary Information [file 41467_2022_34339_MOESM1_ESM.pdf]

Supplementary Information for:

XNAzymes targeting the SARS-CoV-2 genome inhibit viral infection

Pehuén Pereyra Gerber[1], Maria J. Donde[1], Nicholas J. Matheson[1,2,3] and Alexander I. Taylor[1]*

[1]Cambridge Institute of Therapeutic Immunology & Infectious Disease (CITIID), Jeffrey Cheah Biomedical Centre, University of Cambridge, UK.

[2]Department of Medicine, University of Cambridge, UK.

[3]NHS Blood and Transplant, Cambridge, UK.

*corresponding author: ait29@cam.ac.uk

Contents:

Supplementary Figures 1 – 8.
Supplementary Table 1.
Supplementary References.

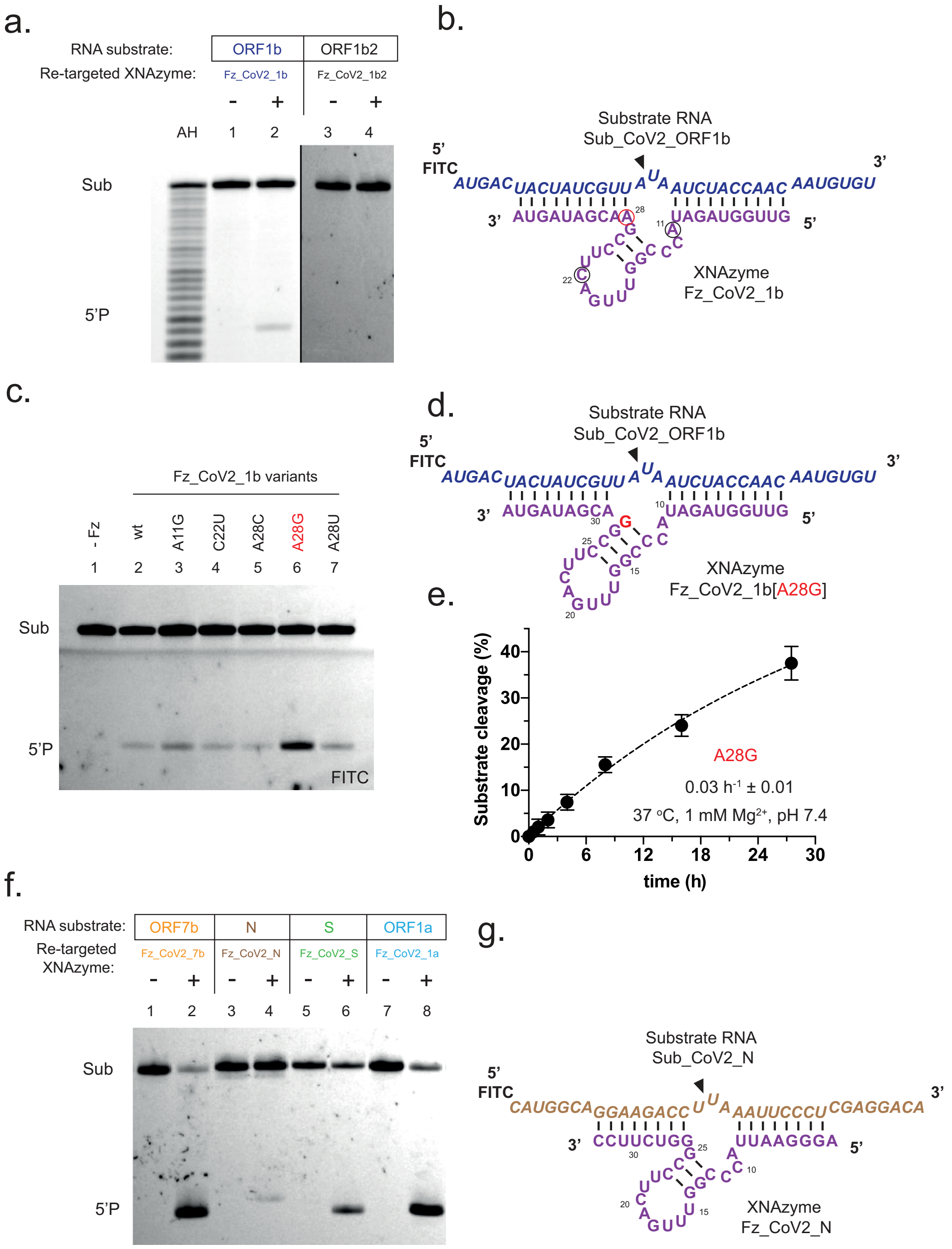

Supplementary Figure 1. Re-targeting RNA endonuclease XNAzymes to sequences in the SARS-CoV-2 genome.

*(**a**) Urea-PAGE gel showing reactions between (1 μM) RNA substrates Sub_CoV2_ORF1b (lanes 1 and 2) or Sub_CoV2_ORF1b2 (lanes 3 and 4), and (5 μM) re-targeted FR6_1 XNAzyme variants Fz_CoV2_1b (lane 2) and Fz_CoV2_1b2 (lane 4), respectively (25 h). (AH) indicates Sub_CoV2_ORF1b subjected to partial alkaline hydrolysis. RNA substrate and 5′ product are indicated by "Sub" and "5′P", respectively. (**b**) Schematic showing sequences and putative secondary structure of Fz_CoV2_1b bound to RNA substrate Sub_CoV2_ORF1b. Purple indicates FANA residues. Black arrow indicates site of substrate cleavage. Mutations of circled positions were previously found to improve activity of a re-targeted FR6_1 variant (see reference [1]); (**c**) Urea-PAGE gel showing reactions between (5 μM) variants of Fz_CoV2_1b corresponding to mutations at these positions and (1 μM) RNA substrate Sub_CoV2_ORF1b (15 h), revealing (**d**) improved variant Fz_CoV2_1b [A28G]. (**e**) Graph showing timecourse of pre-steady state bimolecular reaction of substrate RNA Sub_CoV2_ORF1b (1 μM) with Fz_CoV2_1b [A28G] (5 μM). Error bars show standard error of the mean of three independent replicates. (**f**) Urea-PAGE gel showing reactions between (1 μM) RNA substrates Sub_CoV2_ORF7b (lanes 1 and 2), Sub_CoV2_N (lanes 3 and 4), Sub_CoV2_S (lanes 5 and 6) or Sub_CoV2_ORF1a (lanes 7 and 8), and (5 μM) re-targeted FR6_1 XNAzyme variants Fz_CoV2_7b (lane 2), Fz_CoV2_N (lane 4), Fz_CoV2_S (lane 6) and Fz_CoV2_1a (lane 8), respectively (15 h). (**g**) Schematic showing putative secondary structure of Fz_CoV2_N bound to RNA substrate Sub_CoV2_N. All reactions were performed under quasi-physiological conditions (37 °C, 1 mM Mg^{2+}, pH 7.4). Gels shown are representative of two independent experiments.*

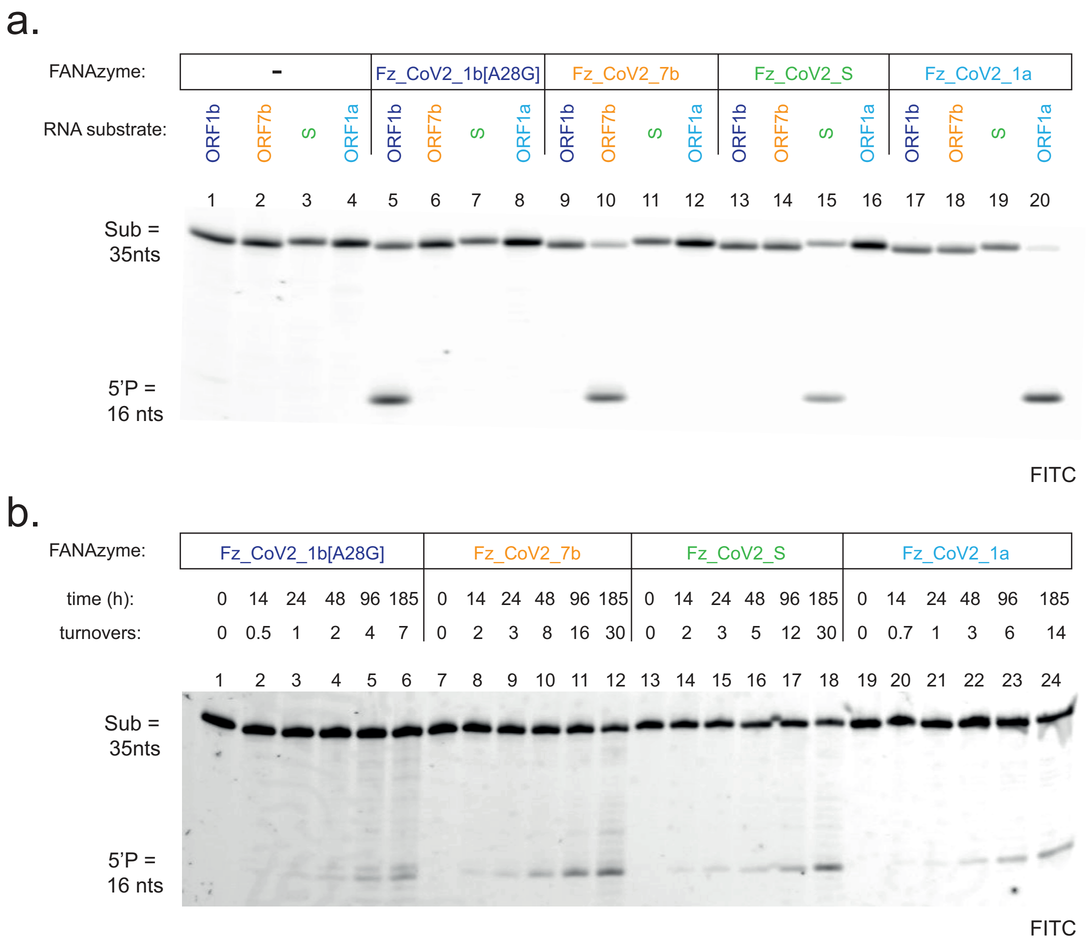

Supplementary Figure 2. Characterization of XNAzymes targeting SARS-CoV-2 RNA.

*(**a,b**) Urea-PAGE gels showing RNA cleavage reactions of the most active SARS-CoV-2 XNAzymes to determine: (a) specificity, by reacting (5 μM) XNAzymes with (1 μM) cognate or non-cognate RNA substrates (48 h), (b) capacity for multiple-turnover catalysis, by reacting (10 nM) each XNAzyme with (1 μM) appropriate cognate RNA substrate for times shown. All reactions were performed under quasi-physiological conditions (37 °C, 1 mM Mg^{2+}, pH 7.4). Gels shown are representative of two independent experiments.*

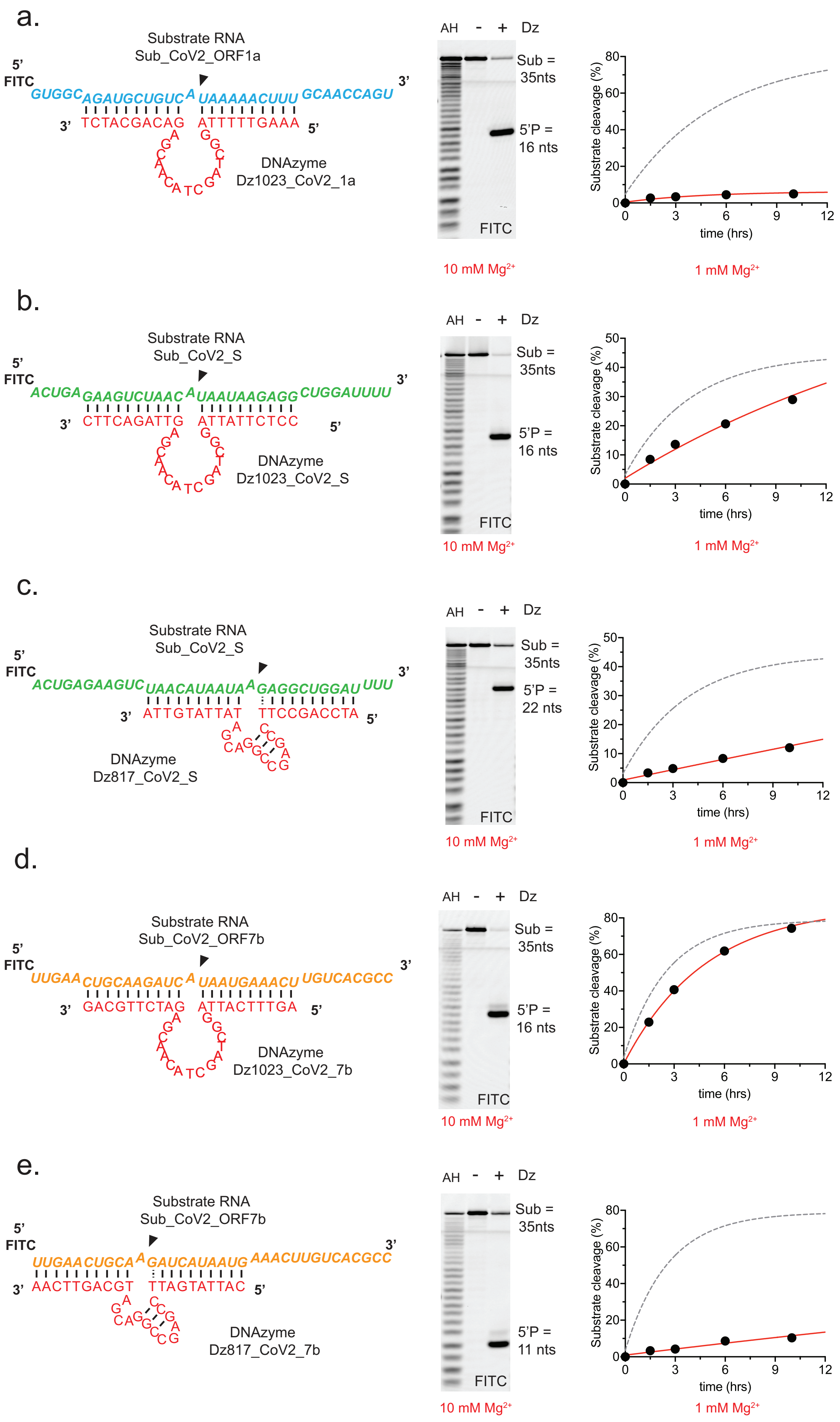
a.
Substrate RNA
Sub_CoV2_ORF1a
5' FITC
GUGGC AGAUGCUGUC A UAAAAACUUU GCAACCAGU 3'
3' TCTACGACAG ATTTTTGAAA 5'
DNAzyme
Dz1023_CoV2_1a
AH - + Dz
Sub = 35nts
5'P = 16 nts
FITC
10 mM Mg2+
Substrate cleavage (%)
time (hrs)
1 mM Mg2+
b.
Substrate RNA
Sub_CoV2_S
5' FITC
ACUGA GAAGUCUAAC A UAAUAAGAGG CUGGAUUUU 3'
3' CTTCAGATTG ATTATTCTCC 5'
DNAzyme
Dz1023_CoV2_S
AH - + Dz
Sub = 35nts
5'P = 16 nts
FITC
10 mM Mg2+
Substrate cleavage (%)
time (hrs)
1 mM Mg2+
c.
Substrate RNA
Sub_CoV2_S
5' FITC
ACUGAGAAGUC UAACAUAAUA A GAGGCUGGAU UUU 3'
3' ATTGTATTAT TTCCGACCTA 5'
DNAzyme
Dz817_CoV2_S
AH - + Dz
Sub = 35nts
5'P = 22 nts
FITC
10 mM Mg2+
Substrate cleavage (%)
time (hrs)
1 mM Mg2+
d.
Substrate RNA
Sub_CoV2_ORF7b
5' FITC
UUGAA CUGCAAGAUC A UAAUGAAACU UGUCACGCC 3'
3' GACGTTCTAG ATTACTTTGA 5'
DNAzyme
Dz1023_CoV2_7b
AH - + Dz
Sub = 35nts
5'P = 16 nts
FITC
10 mM Mg2+
Substrate cleavage (%)
time (hrs)
1 mM Mg2+
e.
Substrate RNA
Sub_CoV2_ORF7b
5' FITC
UUGAACUGCA A GAUCAUAAUG AAACUUGUCACGCC 3'
3' AACTTGACGT TTAGTATTAC 5'
DNAzyme
Dz817_CoV2_7b
AH - + Dz
Sub = 35nts
5'P = 11 nts
FITC
10 mM Mg2+
Substrate cleavage (%)
time (hrs)
1 mM Mg2+

Supplementary Figure 3. Analogous DNAzymes are less active than the SARS-CoV-2 RNA-targeting XNAzymes under quasi-physiological conditions*.*

*(**a-e**) Schematic showing sequences and putative secondary structure of DNAzymes based on the (a,b,d) 10-23 or (c,e) 8-17 catalysts[2,3], designed to target the same SARS-CoV-2 RNA substrates as the three most active XNAzymes, (a) Sub_CoV2_ORF1a, (b,c) Sub_CoV2_S and (d,e) Sub_CoV2_ORF7b; Urea-PAGE gels showing reactions between (1 μM) RNA substrate and (5 μM) DNAzyme (24 h) in high magnesium buffer (37 ^{o}C, 10 mM Mg^{2+}, pH 7.4), (AH) indicates RNA substrate subjected to partial alkaline hydrolysis; graphs showing timecourse of pre-steady state bimolecular reactions between (1 μM) RNA substrate and (5 μM) DNAzyme (black circles and red lines)(dashed grey lines show the rates for the analogous XNAzymes from Fig. 1) in quasi-physiological conditions (37 ^{o}C, 1 mM Mg^{2+}, pH 7.4). Gels shown are representative of two independent experiments.*

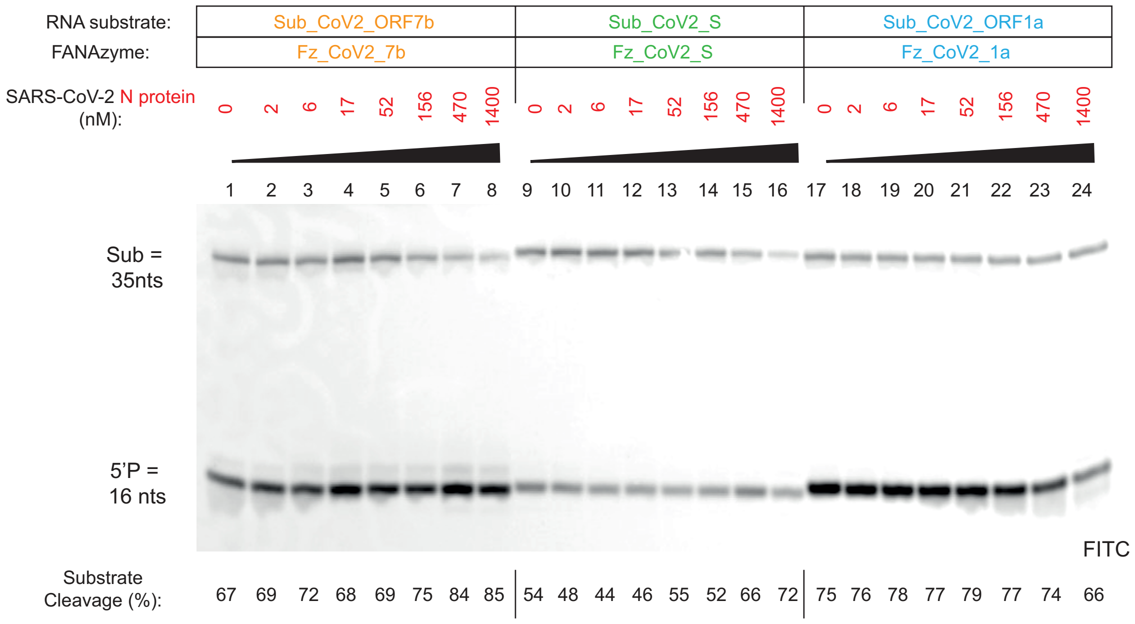

Supplementary Figure 4. Activity of XNAzymes in the presence of recombinant SARS-CoV-2 nucleocapsid (N) protein.

Urea-PAGE gels showing RNA cleavage reactions of the most active SARS-CoV-2 XNAzymes in the presence of SARS-CoV-2 nucleocapsid (N) protein, by reacting (5 μM) each XNAzyme with (1 μM) appropriate cognate RNA substrate, with increasing concentrations of N protein as shown (19 h). All reactions were performed under quasi-physiological conditions (37 °C, 1 mM Mg^{2+}, pH 7.4). Gels shown represents a single experiment.

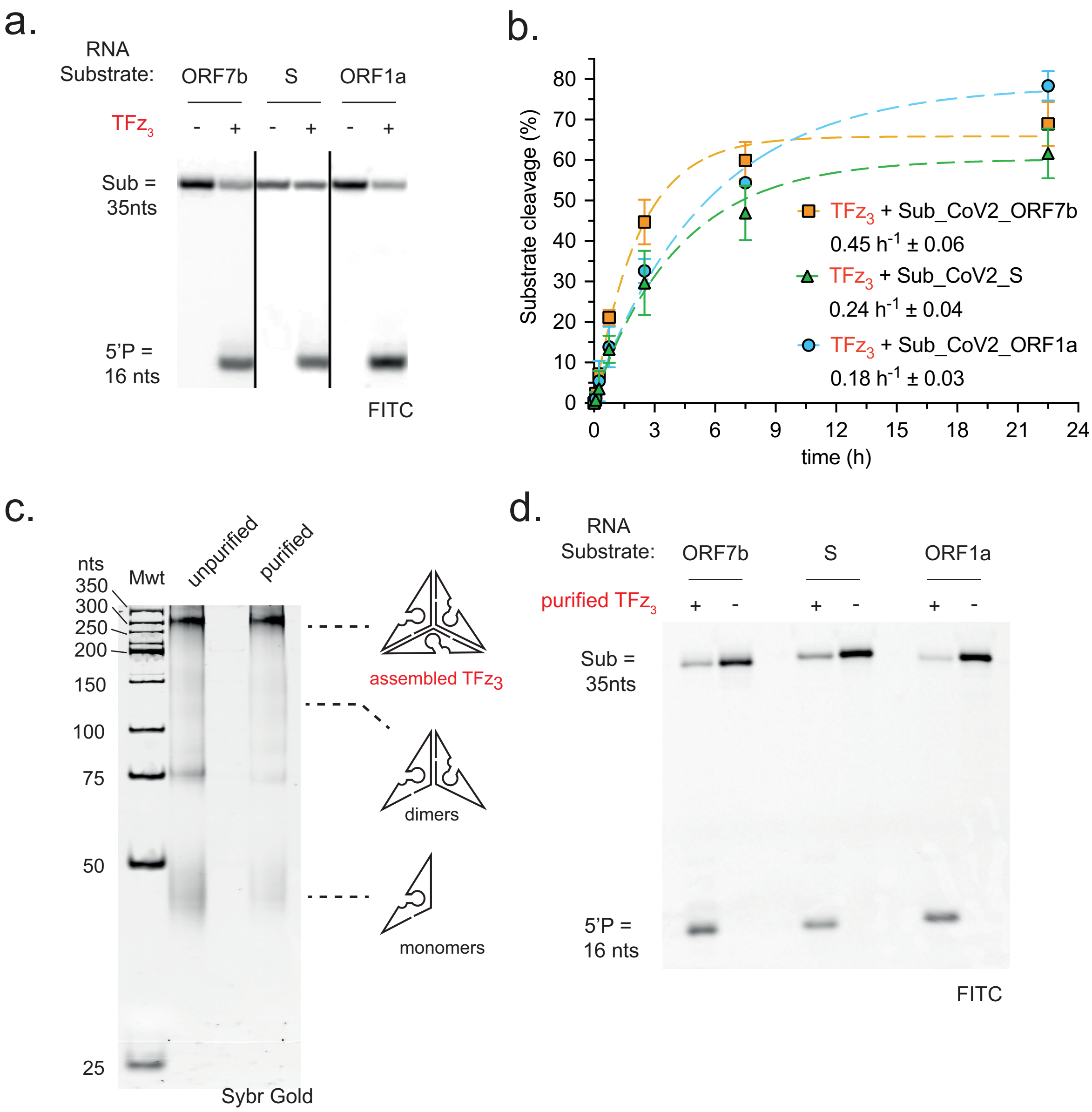

Supplementary Figure 5. XNAzymes targeting SARS-CoV-2 RNA retain full activity when assembled into a nanostructure.

*(**a**) Urea-PAGE gels and (**b**) graph showing timecourses of pre-steady state bimolecular reactions of (1 μM) RNA substrates Sub_CoV2_ORF7b (orange squares), Sub_CoV2_S (green triangles), or Sub_CoV2_ORF1a (cyan circles) with (5 μM) TFz_3 nanostructure (composed of TFz_CoV2_7b, TFz_CoV2_S, TFz_CoV2_1a)(17 h). Data are presented as mean values +/- SEM (error bars), n = 3 independent reactions. All reactions were performed under quasi-physiological conditions (37 °C, 1 mM Mg^{2+}, pH 7.4). (**c**) Native PAGE gel showing preparations of TFz_3 nanostructure before and after purification of the assembled trimeric TFz_3 using a size exclusion spin column to deplete lower molecular weight species corresponding to unassembled monomers and dimers. (**d**) Urea-PAGE gel showing same reactions as (a) using purified TFz_3. Gels shown are representative of two independent experiments.*

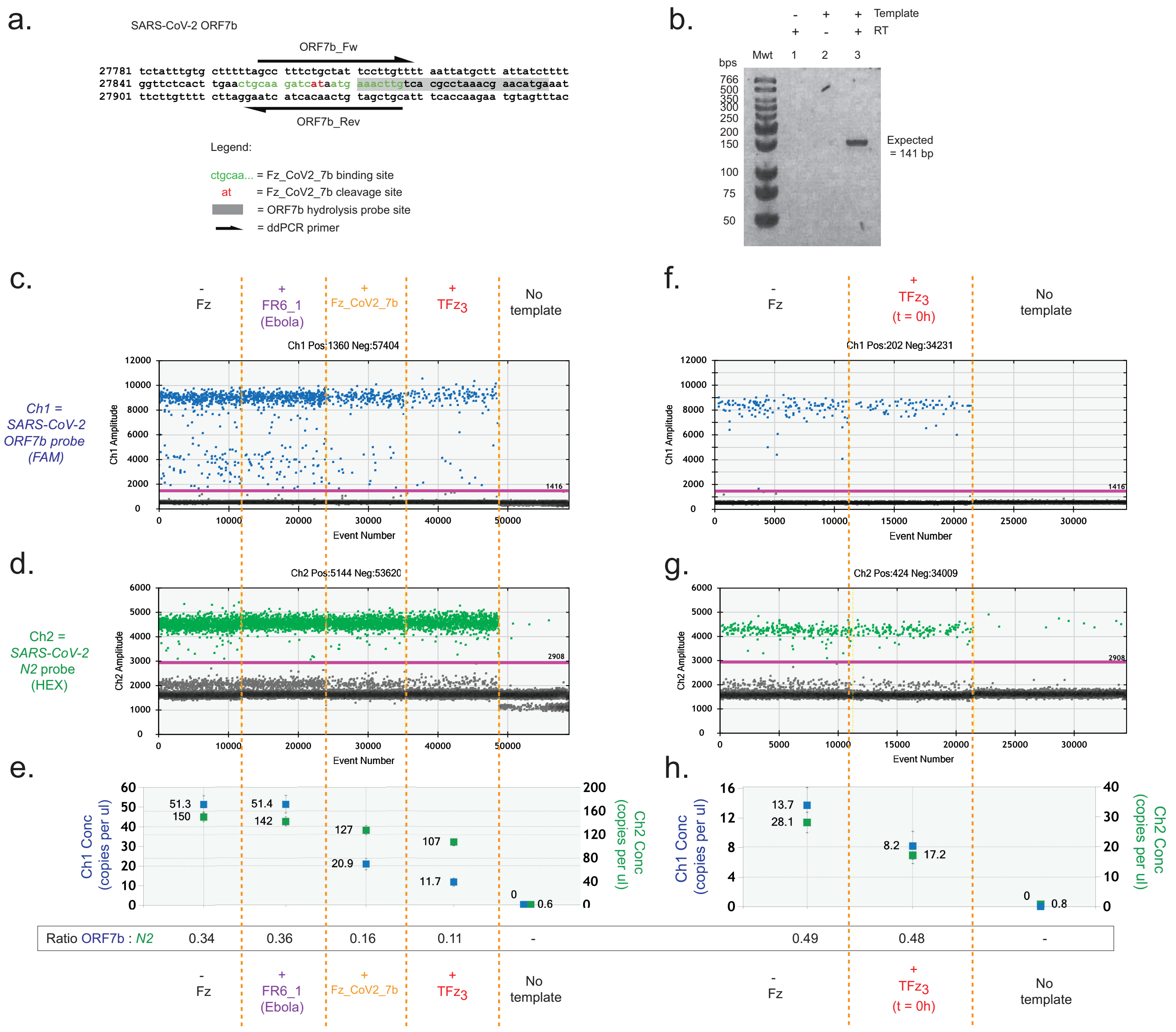

Supplementary Figure 6. A droplet digital RT-qPCR (ddPCR) assay shows XNAzyme-mediated cleavage of ex vivo SARS-CoV-2 genomic RNA.

*(**a**) Residues 27781-27960 of the SARS-CoV-2 genome reference sequence (NCBI NC_045512.2) showing target site of XNAzyme Fz_CoV2_7b in ORF7b, and primers and probe for droplet digital RT-qPCR (ddPCR) assay. (**b**) Qualitative RT-PCR using ORF7b primer set with total RNA extract from SARS-CoV-2 infected cells (kindly provided by S. Baker, University of Cambridge) as template, verifying that the ORF7b assay produces only the expected amplicon (performed using same cycling conditions as ddPCR). Representative of two independent experiments. (**c-h**) Ex vivo SARS-CoV-2 genomic RNA (gRNA) (~3,000 copies/μl) was incubated with (0.5 μM) XNAzyme Fz_CoV2_7b, the catalytic XNA nanostructure (TFz$_3$), an irrelevant XNAzyme (FR6_1B, targeted to a site in the Zaire Ebolavirus genome, see reference [1]), or buffer alone under quasi-physiological conditions (37 °C, 1 mM Mg^{2+}, pH 7.4) in the presence of total cell RNA for (**c-e**) 5 h then purified, or (**f-h**) purified without incubation, reverse transcribed and quantified by ddPCR using (**c** and **f**) the ORF7b assay shown in (a) or (**d** and **g**) the CDC N2 primer and probe set[4] (which detects a site in the nucleocapsid (N) gene that is not targeted by the XNAzymes embedded in TFz$_3$). (**e** and **h**) Summary of quantified SARS-CoV-2 ORF7b-region cDNA copies per μl (Channel 1; blue squares, plotted on left y-axis) or SARS-CoV-2 N-region cDNA copies per μl (Channel 2; green squares, plotted on right y-axis) in the gRNA reactions described above. Ratio of copies per μl in Channel 1 to Channel 2 thus provides a measure of site-specific cleavage by the ORF7b-targeting XNAzyme relative to total gRNA in each reaction.*

*Note that in the non-incubated control reactions (**f-h**) smaller reaction volumes were used, and thus less cDNA in ddPCRs, due to limited supply of SARS-CoV-2 genomic RNA.*

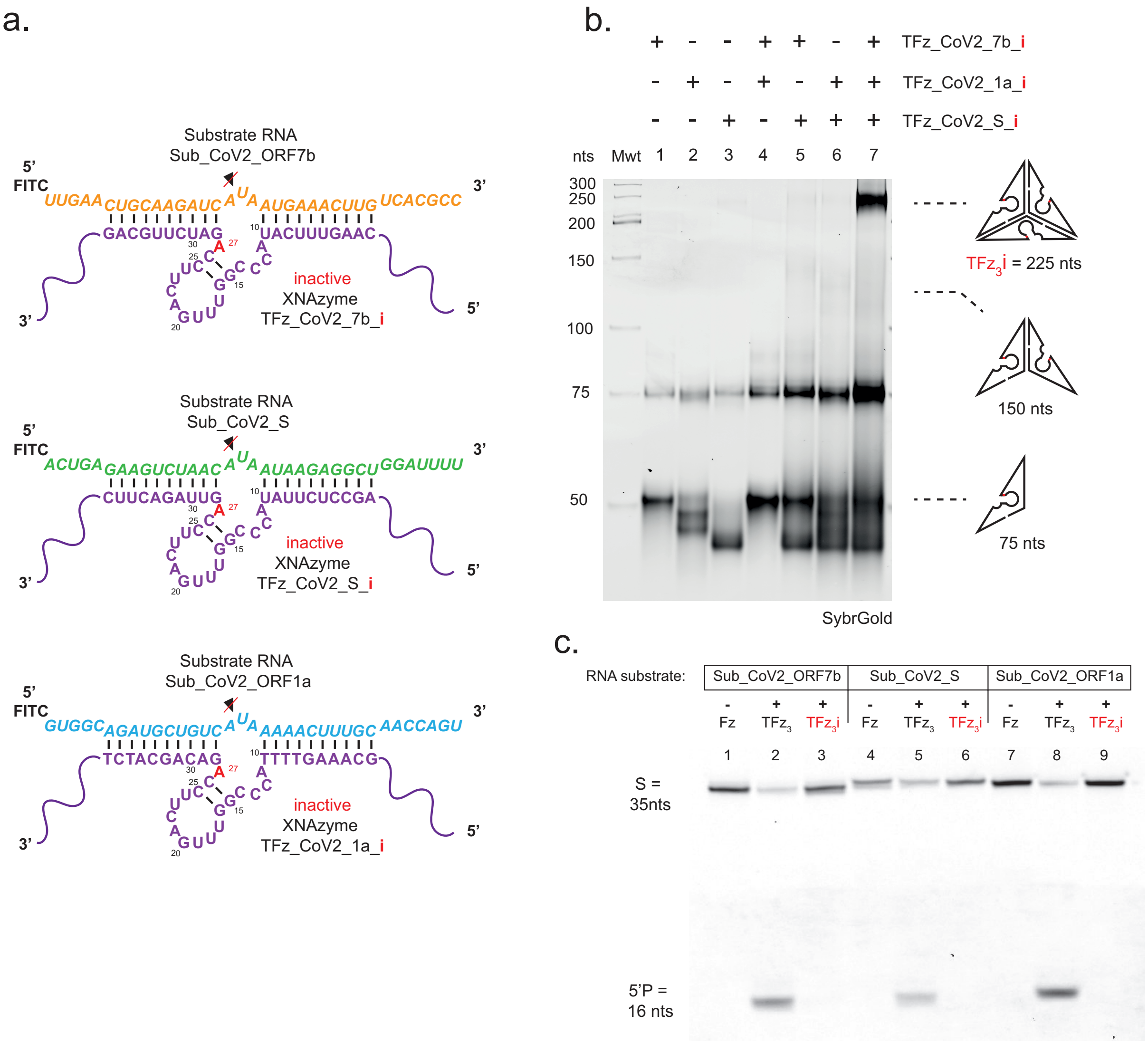

Supplementary Figure 7. A catalytically inactive version of the FANA nanostructure.

*(**a**) Schematic showing sequences and putative secondary structures of FANA nanostructure component strands and their respective RNA substrates, with each embedded XNAzyme carrying a mutation [fG27fA] (highlighted in red), which inactivates the FR6_1 catalytic core. (**b**) Native PAGE gel showing self-assembly of the inactive nanostructure. (**c**) Urea-PAGE gel showing reactions of (1 μM) RNA substrates Sub_CoV2_ORF7b, Sub_CoV2_S or Sub_CoV2_ORF1a with (2.5 μM) active (TFz_3) or inactive (TFz_3i) nanostructures (17 h). All reactions were performed under quasi-physiological conditions (37 °C, 1 mM Mg^{2+}, pH 7.4). Gels shown are representative of two independent experiments.*

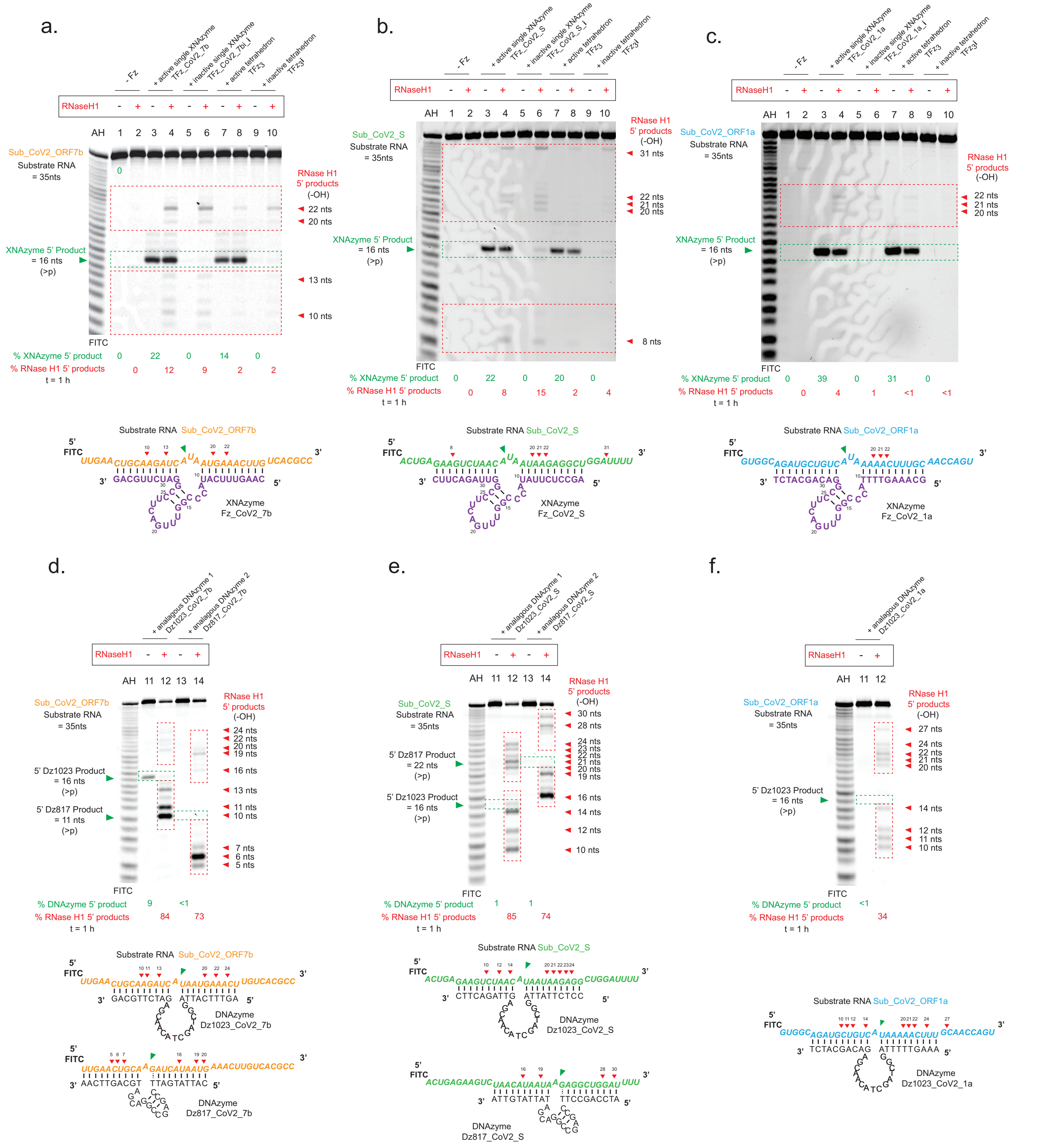

Supplementary Figure 8. Capacity of XNAzymes and DNAzymes targeting SARS-CoV-2 RNA to induce cleavage by RNase H1 in vitro.

*(**a-f**) Urea-PAGE gels showing human RNase H1 assays and schematics showing deduced RNA products and cleavage sites mediated by RNaseH1 (indicated by red dotted line boxes and red arrows) or mediated by XNAzyme or DNAzyme catalysis (indicated by green dotted line boxes and green arrows). Human RNase H1 was incubated with (0.25 µM) RNA substrates (a and d) Sub_CoV2_ORF7b, (b and e) Sub_CoV2_S, or (c and f) Sub_CoV2_ORF1a, and either (a-c) the active (TFz_3) or inactive (TFz_3i; [fG27fA]) versions of the assembled catalytic nanostructure, or the appropriate single-stranded XNAzyme components, or no XNAzyme (-Fz). For comparison, equivalent reactions with (d-f) analogous DNAzymes (see Supplementary Fig. 3) were performed. Note that partial alkaline hydrolysis of RNA substrates (AH), as well as cleavage mediated by the FR6_1 XNAzyme catalytic core, produce 5' RNA products that terminate in 3' cyclic phosphate (>p)[1], whereas RNase H1-mediated cleavage produces 3' OH termini, resulting in a difference in PAGE mobility equivalent to one nucleotide. Gels shown are representative of two independent experiments.*

Supplementary Table 1. Oligonucleotide sequences used in the study.
Black = DNA. Cyan = RNA (FANA sequences were prepared by polymerase using DNA templates shown).

Name	Sequence (5' – 3')	Description
Fz_CoV2_7b_temp	CTGCAAGATCCGGAAGTCAAACCGGGTATG AAACTTGCAATGAGCGATGTGGTAGATT [BiotinTEG]	Template for synthesis of FANAzyme Fz_CoV2_7b
Fz_CoV2_S_temp	GAAGTCTAACCGGAAGTCAAACCGGGTATA AGAGGCTCAATGAGCGATGTGGTAGATT [BiotinTEG]	Template for synthesis of FANAzyme Fz_CoV2_S
Fz_CoV2_1a_temp	AGATGCTGTCCGGAAGTCAAACCGGGTAAA ACTTTGCCAATGAGCGATGTGGTAGATT [BiotinTEG]	Template for synthesis of FANAzyme Fz_CoV2_1a
Fz_CoV2_N_temp	GGAAGACCCGGAAGTCAAACCGGGTAATTC CCTCAATGAGCGATGTGGTAGATT [BiotinTEG]	Template for synthesis of FANAzyme Fz_CoV2_N
Fz_CoV2_1b_temp	CTATCGTTCGGAAGTCAAACCGGGTATCTAC CCAATGAGCGATGTGGTAGATT [BiotinTEG]	Template for synthesis of FANAzyme Fz_CoV2_1b [wt]
Fz_CoV2_1b[A11G]_temp	CTATCGTTCGGAAGTCAAACCGGGCATCTAC CCAATGAGCGATGTGGTAGATT [BiotinTEG]	Template for synthesis of FANAzyme Fz_CoV2_1b with [A11G] mutation
Fz_CoV2_1b[C22U]_temp	CTATCGTTCGGAAATCAAACCGGGTATCTAC CCAATGAGCGATGTGGTAGATT [BiotinTEG]	Template for synthesis of FANAzyme Fz_CoV2_1b with [C22U] mutation
Fz_CoV2_1b[A28C]_temp	ACTATCGTGCGGAAGTCAAACCGGGTATCT ACCCAATGAGCGATGTGGTAGATT [BiotinTEG]	Template for synthesis of FANAzyme Fz_CoV2_1b with [A28C] mutation and 3' binding arm extended by 1nt
Fz_CoV2_1b[A28G]_temp	ACTATCGTCCGGAAGTCAAACCGGGTATCTA CCCAATGAGCGATGTGGTAGATT [BiotinTEG]	Template for synthesis of FANAzyme Fz_CoV2_1b with [A28G] mutation and 3' binding arm extended by 1nt
Fz_CoV2_1b[A28U]_temp	ACTATCGTACGGAAGTCAAACCGGGTATCTA CCCAATGAGCGATGTGGTAGATT [BiotinTEG]	Template for synthesis of FANAzyme Fz_CoV2_1b with [A28U] mutation and 3' binding arm extended by 1nt
Fz_CoV2_1b2_temp	AACAATTTCGGAAGTCAAACCGGGTATTTAC TTCAATGAGCGATGTGGTAGATT [BiotinTEG]	Template for synthesis of FANAzyme Fz_CoV2_1b2
TFz_CoV2_7b_temp	CGTGGGAAGCACATTCCTAAGTCTGAAGTCT GCAAGATCCGGAAGTCAAACCGGGTATGAA ACTTGAAACGACACCAATGAGCGATGTGGT AGATT [BiotinTEG]	Template for synthesis of FANAzyme Fz_CoV2_7b with sequences for nanostructure assembly [analogous to 'S3' in ref [5]]
TFz_CoV2_S_temp	CCATAGTAGATTCCCACGTAGTGTCGTTTGA AGTCTAACCGGAAGTCAAACCGGGTATAAG AGGCTGTGAAGAGCCAATGAGCGATGTGGT AGATT [BiotinTEG]	Template for synthesis of FANAzyme Fz_CoV2_S with sequences for nanostructure assembly [analogous to 'S5' in in ref [5]]
TFz_CoV2_1a_temp	AGGAATGTGTTACTATGGCGGCTCTTCTCAG ATGCTGTCCGGAAGTCAAACCGGGTAAAAC TTTGCGTTTCAGACCAATGAGCGATGTGGTA GATT [BiotinTEG]	Template for synthesis of FANAzyme Fz_CoV2_1a with sequences for nanostructure assembly [analogous to 'S4' in in ref [5]]
TFz_CoV2_7b_i_temp	CGTGGGAAGCACATTCCTAAGTCTGAAGTCT GCAAGATCTGGAAGTCAAACCGGGTATGAA ACTTGAAACGACACCAATGAGCGATGTGGT AGATT [BiotinTEG]	Template for synthesis of FANAzyme Fz_CoV2_7b_i with [fG27fA] mutation and sequences for inactive nanostructure assembly [analogous to 'S3' in ref [5]]

TFz_CoV2_S_i_temp	CCATAGTAGATTCCCACGTAGTGTCGTTTGA AGTCTAACTGGAAGTCAAACCGGGTATAAG AGGCTGTGAAGAGCCAATGAGCGATGTGGT AGATT [BiotinTEG]	Template for synthesis of FANAzyme Fz_CoV2_S_i with [fG27fA] mutation and sequences for inactive nanostructure assembly [analogous to 'S5' in ref [5]]
TFz_CoV2_1a_i_temp	AGGAATGTGTTACTATGGCGGCTCTTCTCAG ATGCTGTCTGGAAGTCAAACCGGGTAAAAC TTTGCGTTTCAGACCAATGAGCGATGTGGTA GATT [BiotinTEG]	Template for synthesis of FANAzyme Fz_CoV2_1a_i with [fG27fA] mutation and sequences for inactive nanostructure assembly [analogous to 'S4' in ref [5]]
FR6_1Btemp	TCAAATTCCGGAAGTCAAACCGGGTATCTAC CACAATGAGCGATGTGGTAGATT [BiotinTEG]	Template for synthesis of XNAzyme FR6_1B (Ebola-specific catalyst used as negative control)
drP2_Ebo	[6FAM]AATCTACCACATCGCTCATTG	Primer for synthesis of XNAzymes (3' residue is RNA to allow primer removal after synthesis)
Sub_CoV2_ORF7b	[6FAM]UUGAACUGCAAGAUCAUAAUGAA ACUUGUCACGCC	Substrate for Fz_CoV2_7b equivalent to nts 27850-27884 in SARS-CoV-2 genome: NCBI reference sequence NC_045512.2
Sub_CoV2_S	[6FAM]ACUGAGAAGUCUAACAUAAUAAGA GGCUGGAUUUU	Substrate for Fz_CoV2_S equivalent to nts 21845-21879 in SARS-CoV-2 genome: NCBI reference sequence NC_045512.2
Sub_CoV2_ORF1a	[6FAM]GUGGCAGAUGCUGUCAUAAAAAC UUUGCAACCAGU	Substrate for Fz_CoV2_1a equivalent to nts 2888-2922 in SARS-CoV-2 genome: NCBI reference sequence NC_045512.2
Sub_CoV2_ORF1b	[6FAM]AUGACUACUAUCGUUAUAAUCUAC CAACAAUGUGU	Substrate for Fz_CoV2_1b equivalent to nts 14798-14832 in SARS-CoV-2 genome: NCBI reference sequence NC_045512.2
Sub_CoV2_ORF1b2	[6FAM]CAUGAAGAAACAAUUUAUAAUUU ACUUAAGGAUUG	Substrate for Fz_CoV2_1b2 equivalent to nts 13684-13718 in SARS-CoV-2 genome: NCBI reference sequence NC_045512.2
Sub_CoV2_N	[6FAM]CAUGGCAAGGAAGACCUUAAAUUC CCUCGAGGACA	Substrate for Fz_CoV2_N equivalent to nts 28448-28482 in SARS-CoV-2 genome: NCBI reference sequence NC_045512.2
eIF2B2_Fw	TCCGGGAGGAGTATGGCAG	Forward primer for eIF2B2 reference ddPCR
eIF2B2_Rev	AATGGAAGCTGAAATCCTCG	Reverse primer for eIF2B2 reference ddPCR
eIF2B2_Probe	[HEX]GCAGGAGTCCCTGCACAAACT [BHQ1]	Hydrolysis probe for eIF2B2 reference ddPCR
nCoV_N2_Fw	TTACAAACATTGGCCGCAAA	Forward primer for SARS-CoV-2 (CDC N2 assay[4]) ddPCR
nCoV_N2_Rev	GCGCGACATTCCGAAGAA	Reverse primer for SARS-CoV-2 (CDC N2 assay) ddPCR
nCoV_N2_Probe	[HEX]ACAATTTGCCCCCAGCGCTTCAG [BHQ1]	Hydrolysis probe for SARS-CoV-2 (CDC N2 assay) ddPCR

nCoV_ORF7b_Fw	GCCTTTCTGCTATTCCTTGTTT	Forward primer for SARS-CoV-2 (ORF 7b assay) ddPCR
nCoV_ ORF7b _Rev	GCAGCTACAGTTGTGATGATTC	Reverse primer for SARS-CoV-2 (ORF 7b assay) ddPCR
nCoV_ ORF7b _Probe	[6FAM]ACTTGTCACGCCTAAACGAACATGA [BHQ1]	Hydrolysis probe for SARS-CoV-2 (ORF 7b assay) ddPCR
Dz1023_CoV2_1a	AAAGTTTTTAGGCTAGCTACAACGAGACAG CATCT	10-23 DNAzyme targeted to Sub_CoV2_ORF1a
Dz1023_CoV2_S	CCTCTTATTAGGCTAGCTACAACGAGTTAGA CTTC	10-23 DNAzyme targeted to Sub_CoV2_S
Dz817_CoV2_S	ATCCAGCCTTCCGAGCCGGACGATATTATGT TA	8-17 DNAzyme targeted to Sub_CoV2_S
Dz1023_CoV2_7b	AGTTTCATTAGGCTAGCTACAACGAGATCTT GCAG	10-23 DNAzyme targeted to Sub_CoV2_ORF7b
Dz817_CoV2_7b	CATTATGATTCCGAGCCGGACGATGCAGTTC AA	8-17 DNAzyme targeted to Sub_CoV2_ORF7b

Supplementary References

1 Taylor, A. I., Wan, J. K., Donde, M. J., Peak-Chew, S.-Y. & Holliger, P. A modular XNAzyme that cleaves long, structured RNAs under physiological conditions enables allele-specific gene silencing in cells. *Nature Chemistry*, doi:10.1038/s41557-022-01021-z (2022).

2 Santoro, S. W. & Joyce, G. F. A general purpose RNA-cleaving DNA enzyme. *Proceedings of the National Academy of Sciences of the United States of America* **94**, 4262-4266 (1997).

3 Faulhammer, D. & Famulok, M. Characterization and divalent metal-ion dependence of in vitro selected deoxyribozymes which cleave DNA/RNA chimeric oligonucleotides. *Journal of molecular biology* **269**, 188-202, doi:10.1006/jmbi.1997.1036 (1997).

4 https://www.cdc.gov/coronavirus/2019-ncov/lab/rt-pcr-panel-primer-probes.html

5 Thai, H. B. D. *et al.* Tetrahedral DNAzymes for enhanced intracellular gene-silencing activity. *Chemical communications (Cambridge, England)* **54**, 9410-9413, doi:10.1039/c8cc05721d (2018).
