# Supplementary material for: XNAzymes targeting the SARS-CoV-2 genome inhibit viral infection
Source: Nat Commun. 2022 Nov 16;13:6716. doi: 10.1038/s41467-022-34339-w (PMC9668987; doi:10.1038/s41467-022-34339-w)
Supplement: Supplementary file 2 — Reporting Summary [file 41467_2022_34339_MOESM2_ESM.pdf]

nature portfolio

Corresponding author(s): Alexander Iain Taylor

Last updated by author(s): Oct 21, 2022

Reporting Summary

Nature Portfolio wishes to improve the reproducibility of the work that we publish. This form provides structure for consistency and transparency in reporting. For further information on Nature Portfolio policies, see our Editorial Policies and the Editorial Policy Checklist.

Statistics

For all statistical analyses, confirm that the following items are present in the figure legend, table legend, main text, or Methods section.

n/a	Confirmed	
☐	☒	The exact sample size (n) for each experimental group/condition, given as a discrete number and unit of measurement
☐	☒	A statement on whether measurements were taken from distinct samples or whether the same sample was measured repeatedly
☐	☒	The statistical test(s) used AND whether they are one- or two-sided<br>*Only common tests should be described solely by name; describe more complex techniques in the Methods section.*
☒	☐	A description of all covariates tested
☒	☐	A description of any assumptions or corrections, such as tests of normality and adjustment for multiple comparisons
☐	☒	A full description of the statistical parameters including central tendency (e.g. means) or other basic estimates (e.g. regression coefficient) AND variation (e.g. standard deviation) or associated estimates of uncertainty (e.g. confidence intervals)
☐	☒	For null hypothesis testing, the test statistic (e.g. F, t, r) with confidence intervals, effect sizes, degrees of freedom and P value noted<br>*Give P values as exact values whenever suitable.*
☒	☐	For Bayesian analysis, information on the choice of priors and Markov chain Monte Carlo settings
☒	☐	For hierarchical and complex designs, identification of the appropriate level for tests and full reporting of outcomes
☒	☐	Estimates of effect sizes (e.g. Cohen's d, Pearson's r), indicating how they were calculated

Our web collection on statistics for biologists contains articles on many of the points above.

Software and code

Policy information about availability of computer code

Data collection	ddPCR: Quantasoft version 1.7.4.0917 (Bio-Rad Laboratories, USA)
Data analysis	ImageQuant TL (GE Heathcare), Fiji / ImageJ2 version 2.3.0/1.53q, Prism 9.2.0 (Graphpad Software, USA)

For manuscripts utilizing custom algorithms or software that are central to the research but not yet described in published literature, software must be made available to editors and reviewers. We strongly encourage code deposition in a community repository (e.g. GitHub). See the Nature Portfolio guidelines for submitting code & software for further information.

Data

Policy information about availability of data

All manuscripts must include a data availability statement. This statement should provide the following information, where applicable:

- Accession codes, unique identifiers, or web links for publicly available datasets
- A description of any restrictions on data availability
- For clinical datasets or third party data, please ensure that the statement adheres to our policy

All the data generated in this study are available within the main text and the Supplementary Information file; source data are provided in the Source Data file. Data are also available from the corresponding author upon request. NCBI SARS-CoV-2 genome reference sequence: NC_045512.2.

Field-specific reporting

Please select the one below that is the best fit for your research. If you are not sure, read the appropriate sections before making your selection.

[x] Life sciences [ ] Behavioural & social sciences [ ] Ecological, evolutionary & environmental sciences

For a reference copy of the document with all sections, see nature.com/documents/nr-reporting-summary-flat.pdf

Life sciences study design

All studies must disclose on these points even when the disclosure is negative.

Sample size	XNAzyme kinetics: n = 3, all timecourses were prepared and measured as biological triplicates. This sample size was chosen to be able to determine pseudo-first order reaction rates by fitting the data. As results from triplicates were consistent, this sample size was deemed to be sufficient.<br>Infection assays: n = 3, all experiments were performed and measured in triplicate, in two independent experiments using two separate batches of cells and XNAzymes. As results from triplicates were consistent, this sample size was deemed to be sufficient.<br>ddPCR: n = 3, all experiments were performed and measured in triplicate, with two technical replicates per sample and at least 10,000 droplets acquired for each reaction. As results between triplicates were consistent, this sample size was deemed to be sufficient.
Data exclusions	No data were excluded from the analyses
Replication	For all results shown in gel scans, the number of times similar, consistent results have been obtained is reported in the respective figure legend. For ddPCR and cell reporter assays, experiments were performed in triplicate and repeated at least twice.
Randomization	Randomisation was not applicable to this study as there was no allocation of samples into experimental groups.
Blinding	Blinding was not applicable to this study as data collection and analysis were not performed in allocated groups

Reporting for specific materials, systems and methods

We require information from authors about some types of materials, experimental systems and methods used in many studies. Here, indicate whether each material, system or method listed is relevant to your study. If you are not sure if a list item applies to your research, read the appropriate section before selecting a response.

Materials & experimental systems

n/a	Involved in the study	
[x]	[ ]	Antibodies
[ ]	[x]	Eukaryotic cell lines
[x]	[ ]	Palaeontology and archaeology
[x]	[ ]	Animals and other organisms
[x]	[ ]	Human research participants
[x]	[ ]	Clinical data
[x]	[ ]	Dual use research of concern

Methods

n/a	Involved in the study	
[x]	[ ]	ChIP-seq
[x]	[ ]	Flow cytometry
[x]	[ ]	MRI-based neuroimaging

Eukaryotic cell lines

Policy information about cell lines

Cell line source(s)	HEK293T reporter cells were generated in the Matheson lab, VeroE6 cells were obtained from Rupert Beale, Francis Crick Institute.
Authentication	HEK293T cells were authenticated by STR profiling, VeroE6 cells were authenticated by species-specific PCR (IDEXX BioAnalytics)
Mycoplasma contamination	Cells were regularly screened and confirmed to be mycoplasma negative (Lonza MycoAlert and IDEXX BioAnalytics).
Commonly misidentified lines<br>(See ICLAC register)	No commonly misidentified lines were used.
